# Detecting artificially impaired balance in human locomotion: metrics, perturbation effects and detection thresholds

**DOI:** 10.1242/jeb.249339

**Published:** 2025-05-22

**Authors:** Jiaen Wu, Michael Raitor, Guan Rong Tan, Kristan L. Staudenmayer, Scott L. Delp, C. Karen Liu, Steven H. Collins

**Affiliations:** ^1^ Department of Mechanical Engineering, Stanford University, Stanford, CA 94305, USA; ^2^ Department of Surgery, Stanford University, Stanford, CA 94305, USA; ^3^ Department of Bioengineering, Stanford University, Stanford, CA 94305, USA; ^4^ Department of Computer Science, Stanford University, Stanford, CA 94305, USA

**Keywords:** Balance, Balance metrics, Perturbation recovery, Artificial impairments, Detection thresholds

## Abstract

Measuring balance is important for detecting impairments and developing interventions to prevent falls, but there is no consensus on which method is most effective. Many balance metrics derived from steady-state walking data have been proposed, such as step-width variability, step-time variability, foot placement predictability, maximum Lyapunov exponent and margin of stability. Recently, perturbation-based metrics such as center of mass displacement have also been explored. Perturbations typically involve unexpected disturbances applied to the subject. In this study we collected walking data from 10 healthy human subjects while walking normally and while impairing balance with ankle braces, eye-blocking masks and pneumatic jets on their legs. In some walking trials we also applied mechanical perturbations to the pelvis. We obtained a comprehensive biomechanics dataset and compared the ability of various metrics to detect impaired balance using steady-state walking and perturbation recovery data. We also compared metric performance using thresholds informed by data from multiple subjects versus subject-specific thresholds. We found that step-width variability, step-time variability and foot placement predictability, using steady-state data and subject-specific thresholds, detected impaired balance with the highest accuracy (≥86%), whereas other metrics were less effective (≤68%). Incorporating perturbation data did not improve accuracy of these metrics, although this comparison was limited by the small amount of perturbation data included and analyzed. Subject-specific baseline measurements improved the detection of changes in balance ability. Thus, in clinical practice, taking baseline measurements might improve the detection of impairment due to aging or disease progression.

## Teaser

**Highlighted Article:** Step-width variability, step-time variability and foot placement predictability are better at detecting impaired balance than maximum Lyapunov exponent and margin of stability metrics when using steady-state walking data.

## INTRODUCTION

Falls are a large problem facing society. Nearly one in three adults over 65 fall each year and many of these falls result in injury, permanent disability or death (Kakara et al., 2023). In addition to causing significant suffering to individual fallers, the prevalence of falls is a problem for public health in general, with falls costing the U.S. healthcare system $50 billion each year (Florence et al., 2018). Understanding falls and developing ways to prevent them is a pressing problem.

An accurate measure of intrinsic balance ability could help to prevent falls. Falls are caused by multiple interacting factors that can broadly be categorized into two groups: lifestyle factors and intrinsic ability. Lifestyle factors include the activities people engage in (e.g. walking for exercise, gardening or climbing stairs), how they do those activities (e.g. walking more slowly or using a stairway handrail) and the environments they navigate (e.g. uneven pavement or icy sidewalks) (Campbell et al., 1990; Rapp et al., 2012; Tarcea et al., 2024). We consider someone's intrinsic balance ability to be their ability to prevent themselves from falling in a specific scenario. Lifestyle factors and intrinsic balance ability interact to contribute to a fall. For example, a person with severely impaired balance who almost never gets out of bed may almost never fall, while someone with excellent intrinsic balance ability may fall during adventurous activities. Programs focused on educating individuals at risk of falls about how to alter their behavior or environment to reduce the likelihood of a fall can be effective (Burns et al., 2022). Accurate measurement of balance ability could help identify individuals who could most benefit from an intervention. Balance ability can be improved with perturbation training (Pai et al., 2014) and some mobility aids (Bateni and Maki, 2005). Accurate measures of balance ability could aid prescription and help researchers develop more effective training protocols and devices.

There are many existing candidate metrics of intrinsic balance ability. These metrics are based on steady-state walking, transient behavior while recovering from a walking perturbation or performance on a battery of movement tasks. The balance metrics developed for analyzing steady-state walking broadly fall into three categories based on variability, sensitivity to initial conditions and simple models of gait.

The measures of balance based on variability can be motivated by reasoning from a simple model of human motion. Human motion can be modelled as a simple closed-loop control architecture with actuators, sensory feedback and control signals. Gait variability could arise from impaired control of the body through any of these factors such as an impaired ability to actuate the limbs, missing sensory information, or sensorimotor signal noise. Any of these sources of impairment could result in larger or more frequent corrections to keep upright, which may be detected in measures of variability. Step-width variability and step-time variability during gait have been measured in many previous studies and overall, the data support a relationship between these measures and balance ability (Ayoubi et al., 2014; Brach et al., 2005, 2007; Hausdorff et al., 2001; Heitmann et al., 1989; Maki, 1997; Moe-Nilssen and Helbostad, 2005; Owings and Grabiner, 2004; Svoboda et al., 2017). However, experimental setup constraints in many of these studies made it infeasible to collect the 400 steps required for accurate variability estimation (Desmet et al., 2019; Owings and Grabiner, 2003). Additional work is needed to increase the certainty of the connection between variability and balance ability using more steps per subject.

Foot placement predictability is conceptually similar to step-width variability, but the differences in calculation of the two metrics may produce different results when measuring balance. Foot placement predictability is calculated using the difference between measured foot position and the foot position predicted by center of mass motion, thereby controlling for the portion of foot placement variability explained by the center of mass motion, applicable to both steady-state and perturbed walking (Wang and Srinivasan, 2014; Joshi and Srinivasan, 2019). Although less established than step-width variability, previous work has connected foot placement control to balance ability (Mahaki et al., 2023). Since this metric has the potential to control for foot placement variability that is unrelated to balance impairment, it could increase the signal-to-noise ratio of the metric compared with step-width variability.

The maximum Lyapunov exponent describes how quickly a system with a given initial condition will diverge from a slightly different initial condition. This measure was developed for systems with accurate models and state measurement, but by using repeated measurements and a reasonable definition of system state, it can be approximated with experimental data (Rosenstein et al., 1993). Previous work has used this approximated maximum Lyapunov exponent to study gait and stability (Bruijn et al., 2009; Buzzi et al., 2003; Dingwell and Cusumano, 2000; Dingwell and Marin, 2006; Dingwell et al., 2000, 2001; Kyvelidou et al., 2008; Manor and Li, 2009; Toebes et al., 2012). The approximated maximum Lyapunov exponent is sometimes referred to as a measure of the ‘local dynamic stability’ in gait research, but we will refer to it as the Lyapunov exponent in this paper to increase the precision of the wording and avoid confusion with other balance metrics.

The margin of stability is a metric derived from a simple model of walking and is often used to assess balance. It is calculated by modelling the body as an inverted pendulum, extrapolating where the center of mass will be in the near future, and finding the distance between this ‘extrapolated center of mass’ and the edge of the foot (Hof et al., 2005). This distance between the edge of the foot and the extrapolated center of mass is defined as the margin of stability. Previous work has characterized margin of stability in several patient populations, such as older adults (Hurt and Grabiner, 2015), and people with stroke (Lencioni et al., 2021; Tisserand et al., 2018), lower-limb amputation (Gates et al., 2013; Rodrigues et al., 2021) and multiple sclerosis (Lencioni et al., 2021; Peebles et al., 2016). While margin of stability has reflected differences between groups in these studies, interpreting the results from margin of stability can be challenging because groups at higher risk of falling are often given scores indicating they are more stable than groups not at risk of falling. Previous studies have suggested the margin of stability may reflect attempts to compensate for impaired balance, rather than the underlying balance ability (Gates et al., 2013; Rethwilm et al., 2021; Rodrigues et al., 2021; Tisserand et al., 2018).

Perturbation-based measures of human balance are motivated by the idea that external disturbances to a system reveal important information about system dynamics and stability. This is common practice in system identification for dynamical systems analysis (Perreault et al., 1998). Additionally, many falls in older adults occur as a result of unexpected changes in contact with the environment (Robinovitch et al., 2013), therefore responses to unexpected perturbations may be the most appropriate conditions under which to study balance. Using perturbations to study human balance is a newer approach than observing steady-state walking. Many candidate signals of balance during perturbation recovery have been explored, such as center of mass motion (Fallahtafti et al., 2023; Vlutters et al., 2016, 2018), foot placement (Kwon et al., 2023; Vlutters et al., 2016, 2018), arm motion (Bruijn et al., 2010, 2022) and whole body angular momentum (Sheehan et al., 2015; Smith et al., 2024), but defining a perturbation-based method for assessing balance ability is still an active area of research. Center of mass displacement following a perturbation (Kwon et al., 2023) is probably the only balance metric that was originally developed for perturbed walking.

Combining perturbations with existing metrics originally developed for steady-state walking may improve impaired balance detection since perturbations may reveal additional information about balance. Relatively few studies examine the effect of perturbations on these measures (Martelli et al., 2016; McAndrew Young et al., 2012).

Another approach to studying balance is to challenge an individual's balance not through perturbations while walking, but by asking them to perform a battery of tasks that are important for daily living and challenge their balance. One popular test is the short physical performance battery (SPPB), which assesses the subject's ability to complete sit-to-stand transitions, stand with feet in different positions, and walk a short, fixed distance. The SPPB is arguably the most successful balance metric, because it has been shown to correlate with falls in a large, retrospective study (Veronese et al., 2014) and to predict falls in a large, multi-year prospective study (Welch et al., 2021).

The SPPB accurately detects the large differences across cohorts in these studies but is limited to detecting more severe gait impairments. The cohorts in these studies had significant mobility impairment with even the healthy cohorts, including people who had difficulty walking or climbing a single flight of stairs (Welch et al., 2021) and those who had fallen in the past year (Veronese et al., 2014). Even with these more impaired cohorts, many of these subjects still received a very healthy score saturating or nearly saturating the scale (Welch et al., 2021). The fact that the SPPB assigned such impaired individuals the healthiest score possible suggests that this metric lacks the sensitivity to detect less impaired individuals who may still benefit from balance interventions.

Evaluating the effectiveness of the established metric for detecting balance disorders often involves the use of thresholds. These thresholds do not inherently convert continuum metrics into binary outcomes but instead serve as reference points to interpret the degree of impairment. Previous work using step-width variability and SPPB applied the same detection thresholds across a cohort one (Brach et al., 2005; Veronese et al., 2014; Welch et al., 2021). Using a single threshold for all members of a cohort to detect impaired balance is convenient because it only requires a single measurement for each subject but is limited in that it effectively compares each subject to the population average, which does not account for inter-subject differences unrelated to balance ability. To address this, biomechanics research often controls for inter-subject differences to examine changes within a subject (Peterson et al., 1985). Similarly, statistical approaches frequently treat the same subject as a ‘control’ for other conditions to increase sensitivity (Shieh and Jan, 2004). Given the precedence in biomechanics and the potential for increased detection sensitivity, exploring the efficacy of subject-specific and cohort-based thresholds can help to improve the sensitivity and applicability of balance metrics for detecting impaired balance.

Balance can be studied by examining real patient populations and healthy control groups or by artificially impairing balance in an experimental session. Real patient impairments often introduce variability owing to the complexity of their conditions, making direct comparisons challenging. Artificial impairments allow for controlled experimentation, where specific factors can be systematically manipulated and studied. This controlled approach could minimize variability and enables within-subject comparisons. Balance impairments can manifest in many ways. Previous studies have used joint-locking braces to restrict actuation (Vlutters et al., 2018; Vlutters et al., 2019), ankle exoskeletons to interfere with ankle push-off (Wang et al., 2024), visual perturbations to interfere with body state estimation (Francis et al., 2015; Franz et al., 2015) and pneumatic jets on the legs to inject noise into foot placement (Segal and Klute, 2014). These studies suggest that using artificial impairments targeting actuation, sensory inputs and signal noise may be an effective way to study balance.

The purpose of this study was: (1) to evaluate whether subject-specific thresholds improve the detection of impaired balance compared with cohort-based thresholds; (2) to examine whether active perturbations enhance the sensitivity of balance impairment detection; and (3) to compare the efficacy of established balance metrics for detecting impaired balance. We recruited healthy human subjects, artificially impaired their balance and applied unexpected perturbations in some walking trials. We compared the accuracy of detecting impaired balance when using subject-specific versus cohort-based thresholds and perturbation recovery motion versus steady-state walking. We assessed the sensitivity of various metric in detecting balance impairments. We expected the results of this study to inform future research on impaired balance detection and facilitate the development of interventions to improve balance.

## MATERIALS AND METHODS

In this study, we recruited 10 healthy human subjects and impaired their balance using three conditions: ankle braces, eye-blocking masks and pneumatic jets. Subjects walked on a treadmill and in some trials we actively challenged their balance ability by applying mechanical perturbations to their pelvises. Using subject motion data from steady-state and perturbation recovery, we evaluated popular balance metrics to assess their ability to classify a condition as impaired or unimpaired. We tested six metrics: step-width variability, step-time variability, foot placement predictability, Lyapunov exponent, lateral margin of stability and center of mass displacement. We also evaluated impairment detection accuracy when using a subject-specific threshold compared with using a single cohort-based threshold for all subjects.

### Data collection

#### Subject information

Ten healthy individuals (*N*=10; age=24–31 year; mass=68±13 kg; height=1.71±0.09 m; 4 female, 6 male) participated in the study. Subjects provided written informed consent prior to participation. The study design was approved by the Stanford Institutional Review Board (IRB-57846).

#### Impairment conditions

**Fig. 1.**
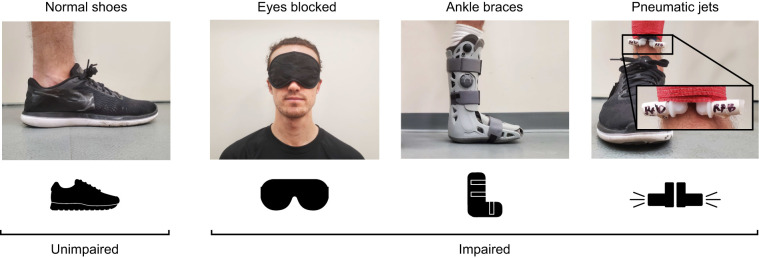
**Artificial impairments to human locomotion.** ‘Normal shoes’ condition is considered unimpaired whereas ‘eyes blocked’, ‘ankle braces’ and ‘pneumatic jets’ are considered impaired conditions. Photograph of pneumatic jets condition has enlarged callout showing pneumatic jet nozzles.

Each subject experienced one unimpaired balance condition and three impaired conditions: ankle braces, eye-blocking masks and pneumatic jets (Fig. 1). The unimpaired condition was intended to serve as a control condition, with subjects wearing standard athletic shoes as they walked on the treadmill. The first impairment condition consisted of wearing Aircast ankle braces (Vista, CA, US) on both legs. This condition was intended to restrict ankle movement and emulate a neuromuscular disorder that primarily impairs balance through reduced dimensions of control, such as reduced strength of ankle plantarflexors (Vlutters et al., 2018, 2019). The second impairment condition consisted of an eye mask that completely blocked the subject's vision. This condition was intended to emulate a balance impairment caused through reduced sensory information, such as a visual impairment. The third impairment condition consisted of pneumatic jets worn on the distal end of both shanks, just above the ankle joint. The jets pointed in the medial and lateral directions and applied force via bursts of air beginning at 0%, 25%, 50% or 75% of the swing phase and lasting between 25 and 100% of the swing phase in duration. The onset timing, duration and direction were pseudorandomized so that subjects could not anticipate the disturbance and compensate for it. The pneumatic disturbance at each step was modulated using onset time since we used a relatively constant pressure source (100–120 psi) and released the pressurized air via a solenoid valve with binary states of ‘open’ or ‘closed’. This system produced a force of approximately 8.8 N. This pneumatic system was based on previous work using pneumatic jets to disrupt normal walking (Segal and Klute, 2014). Because the pneumatic jets produced considerable noise, subjects wore ear protection during this condition. The pneumatic jets condition was intended to emulate a condition resulting in elevated neuromuscular signal noise, such as aging. The order in which each subject experienced the impairment conditions was pseudorandomized across subjects to block against potential ordering effects.

#### Perturbation information

**Fig. 2.**
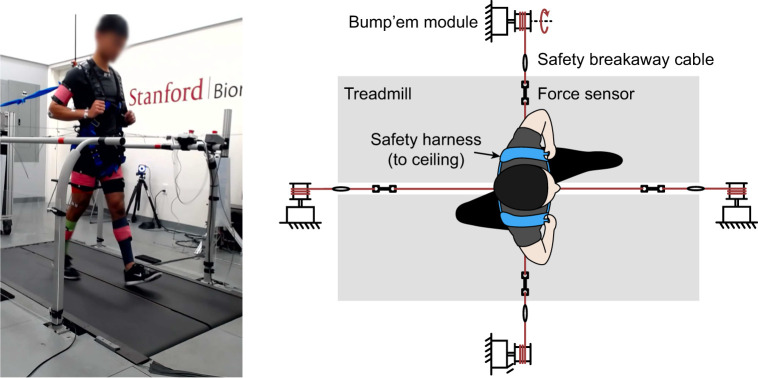
**Experimental setup.** Left: photograph of subject walking on treadmill while wearing safety harness, perturbation harness, motion capture markers and EMG sensors. Subject provided permission for photograph use and their face is blurred to preserve anonymity. Right: top-down view of schematic representing a subject walking on a treadmill while wearing a safety harness and connected to the perturbation system.

In some trials, mechanical perturbations were applied to the subject's pelvis as they walked on the treadmill at 1.25 ms^−1^. Each subject wore a harness around their pelvis that was connected to the Bump'em modules (Tan et al., 2020) distributed around the treadmill. Each perturbation module was connected to the harness via a rope with a force sensor in series to measure the force applied to the subject (Fig. 2). The ropes were connected to the harness such that the line of action of the force through the rope passed through the approximate center of the pelvis. Because the ropes connected to both sides of the harness subjects were not able to swing their arms freely. To prevent different adaptations to this constraint among subjects and to ensure their arms were not caught in the rope, subjects were instructed to walk with their hands loosely holding onto shoulder straps. The perturbation system is described in greater detail in a previous publication and open-source project website (Tan et al., 2020).

To obtain the most useful data from perturbation recovery metrics, it is important that the perturbation is unexpected. If a subject can anticipate a perturbation, their response may be meaningfully different than if the perturbation is unexpected (Pater et al., 2015). To ensure unpredictability, the perturbations applied to subjects occurred in a variety of magnitudes and directions at unexpected times. The perturbations were delivered to the subjects' pelvises with either 7.5% or 15% bodyweight magnitude for a duration of 300 ms. They were applied in the forward, backward, rightward or leftward directions, and began at midstance of the left leg. This combination of four directions and two magnitudes resulted in eight unique perturbation types. Each of these perturbation directions and magnitudes were repeated twice and applied in a pseudorandom order. Perturbation order was pseudorandomized across conditions within the same subject to prevent an individual subject learning the perturbation order and developing anticipatory responses. Perturbations occurred 25–30 s apart sampled from a uniform distribution to prevent the user from anticipating perturbation onset time. Motion was recorded from 10 s before a perturbation onset until 11 s after.

Real-time stance estimation was done using the instrumented split-belt treadmill, which has a separate force plate under the left and right belts. When not recovering from a perturbation, subjects' feet typically landed on the appropriate force plates. This separate measurement of foot contact enabled clean detection of each stride using vertical ground reaction forces. The ground reaction forces were low-pass filtered with a second-order Butterworth filter with a 60 Hz cutoff frequency. We detected heel strike using the rising edge of the force signal crossing a threshold of 100 N and we detected toe-off using the falling edge of the force signal crossing a threshold of 100 N. Using a sliding average of the stride time over 20 strides, we estimated the average stride time and triggered the perturbation system to reach the desired perturbation force at 32.5% of the left leg gait cycle. This timing was chosen based on pilot testing, which indicated that mid-stance perturbations at this point elicited the most distinct differences between impaired and unimpaired conditions. No crossover steps or steps on both belts simultaneously occurred in this dataset because of the haptic boundary setup described below. Future work could improve real-time stance detection by integrating intelligent algorithms that account for crossover steps, utilizing center of pressure signals for more precise gait phase identification.

We used the perturbation system to render a haptic boundary around subjects while they were on the treadmill to ensure subject safety. In order to ensure subjects did not bump into the handrails on either side of the treadmill or walk off the front or back of the treadmill while recovering from a perturbation, a haptic boundary was rendered around the perimeter of the treadmill. This boundary was approximately 0.4 m long (in the anterior–posterior direction) and approximately 0.3 m wide (in the medial–lateral direction) centered at the middle of the treadmill. The exact size of the haptic boundary was set based on user waist measurements to give them the maximum amount of space to move around on the treadmill while maintaining their safety. The haptic boundary was parameterized as a spring-damper system with a damping ratio of 0.13 and a 5 cm ramp length from zero resistance up to a saturation point of 15% bodyweight. This constant 15% bodyweight force continues for 3 cm before a subject would reach the handrails with their pelvis. This damping, spring stiffness and buffer space was set to ensure the boundary was strong enough and had a gentle onset when activated. During the eyes-blocked condition, subjects could not use vision to guide themselves to be near the center of the treadmill. To help subjects stay near the center of the treadmill before a perturbation in the eyes-blocked condition, the haptic boundary was shrunk to a 0.04 m×0.04 m square in the center of the treadmill, which was small enough to keep them centered and large enough to allow comfortable walking. This small boundary modestly restricted pelvis motion during steady-state walking so the boundary was immediately returned to the original size as soon as a perturbation began to avoid interfering with the effects of the perturbation. The boundary was rarely activated during perturbation recoveries for all conditions except the eyes-blocked condition.

#### Measurements

We collected human subject motion, reaction forces and muscle activity to enable our own analysis of balance and enable future studies to make use of this dataset. Subjects walked on an instrumented treadmill with a walking surface of 2 m in length and 1.1 m in width (Bertec, Columbus, OH, USA). Subject motion was recorded using an 11-camera optical motion capture system recording at 100 Hz (Vicon Motion systems, Yarnton, UK). To record subject motion, we used a Plugin gait model marker set with additional markers on the upper arms, thighs, and shanks. Marker trajectory gaps were filled with spline and rigid body assumptions. Treadmill ground reaction forces and moments and perturbation system forces were captured at 1000 Hz. Subjects wore bipolar electromyography (EMG) sensors recording at 1000 Hz (Delsys, Natick, MA, USA), placed on eight muscles of each leg, including the gluteus maximus, gluteus medius, rectus femoris, vastus lateralis, semitendinosus, medial gastrocnemius, soleus and tibialis anterior. All signals were time-synchronized using a field-programmable gate array (Speedgoat, Liebefeld, Switzerland). Note that this study focused on analyzing kinematic and kinetic data. EMG data were collected to build a comprehensive dataset for potential use in future studies, it was not used in the metrics analyses presented in this work.

#### Trial order

**Fig. 3.**
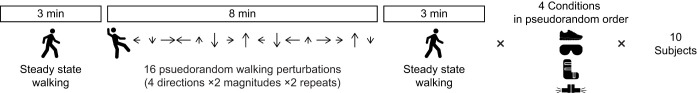
**Experiment protocol.** In each condition, each subject experienced approximately 3 min of walking, followed by 16 perturbations over approximately 8 min, and finally, 3 min of steady-state walking. Perturbation order was pseudo-randomized across conditions within a subject and across subjects. Order in which conditions were experienced were also pseudorandomized across subjects.

In each balance condition, subjects performed the same set of calibration and motion trials. Dynamic motion capture calibration and maximum voluntary muscle contraction trials were recorded prior to beginning to walk on the treadmill to collect the data of interest. The treadmill was set to 1.25 ms^−1^ for all walking trials. Each subject first walked for approximately 3 min in steady-state walking (without perturbations) and were told they would not receive perturbations during this trial. Following this unperturbed walking trial, subjects were alerted that they would be receiving perturbations during the upcoming trial and then received the 16 pseudorandomly ordered perturbations, as described in the ‘Perturbation information’ section (Fig. 3). Following the perturbation bout, subjects were told they would not receive any more perturbations and then walked for approximately 3 min of steady-state walking. In total, subjects walked for 1 h and experienced 64 perturbations. Subjects experienced all conditions and perturbations on the same experiment day to facilitate subject recruitment.

#### Safety features

Safety was a major focus of the design of our experimental setup, and we used a multi-faceted approach leveraging safety features at the electrical, mechanical, software and procedural level. We included emergency stop buttons that stopped electrical power to the treadmill, perturbation system and pneumatic system. Subject emergency stop buttons were placed on the treadmill handles and experimenter buttons were placed on the desk near the control computer. We included mechanical breakaway cables that acted as mechanical fuses, separating the perturbation system from the subject in the event that too much force was applied (Fig. 2). Each subject also wore a safety harness that was tethered to the ceiling so that if the subject lost their balance, they would not hit the floor if they started to fall. We implemented software limits that triggered a fault state disabling all systems if any of the sensor readings moved outside their normal range or if the subject stepped off the treadmill. We also used an experiment checklist that ensured all safety systems were checked before and during each stage of the experiment.

### Balance metric evaluation

#### Perturbation versus steady-state data evaluation

We hypothesized that more accurately recreating the scenarios that contributed to a fall would increase the accuracy of in-laboratory assessments of balance and thereby increase the ability to distinguish unimpaired and impaired balance using existing metrics. Many falls occur as a result of unexpected changes in contact with the environment (Robinovitch et al., 2013), so we evaluated existing balance metrics using only steady-state data as well as either perturbation recovery data only or steady-state plus perturbation recovery data, depending on the metric. For the analysis of step-width variability, step-time variability, foot placement predictability, Lyapunov exponent and lateral margin of stability metric, both steady-state data and steady-state plus perturbation recovery data were used. Those metrics were originally designed for steady-state walking, we incorporated combined datasets to assess their sensitivity to recovery dynamics following perturbations. For analysis of the perturbation-specific metric center of mass displacement, steady-state data and perturbation recovery data were used separately. Comparing metric performance between using only steady-state data and either perturbation recovery data only or steady-state plus perturbation recovery data, depending on the metric, allows us to assess if including perturbation recovery data in assessing balance with existing metrics is beneficial. Owing to the differences in balance metrics evaluated in this study, the subset of steady-state and perturbation recovery data used differs in each metric evaluation and is described in detail in the subsection describing each metric.

#### Subject-specific and cohort-based thresholds

Historically, many balance metrics have been evaluated using cohort-based threshold detection models (Brach et al., 2005). Since using a cohort-based approach essentially uses the mean performance of the group to separate unimpaired and impaired subjects, the approach is not able to consider inter-subject variability unrelated to the impairment of interest, which can be substantial in biomechanical measurements. This cohort-based thresholding works well when the impairment is large relative to the inter-subject variability, and does work for detecting balance impairment in people with severe balance impairment (Veronese et al., 2014; Welch et al., 2021). We implemented binary classification using logistic regression to assess cohort-based detection of balance impairments in our data. The logistic regression problem was set to classify the normal shoes condition as unimpaired and the three balance impairments as impaired. Owing to the imbalance in the number of datapoints between the two classes, we used weighted logistic regression to balance the weighting of the data and make ‘chance performance’ of the classifier 50% accuracy. The inputs to the logistic regression were scores output by a given balance metric (e.g. step-width variability) and the model output was a binary label of ‘impaired’ or ‘unimpaired’. We used leave-one-subject-out cross-validation on all 10 subjects and evaluated the accuracy of the labels assigned to the test subject. The test accuracy across all 10 folds were averaged and that was the detection accuracy used to assess cohort-based thresholds for each balance metric.

To improve sensitivity to less severe impairments and block against natural differences among subjects, we also chose to evaluate how using changes in measurements within the same subject improves impaired balance detection. There are several ways to think about this calculation. One interpretation is that it checks whether a given metric correctly identifies that an impaired condition for subject 1 is more impaired than the unimpaired condition for subject 1 (e.g. impaired score worse than unimpaired score). Another way to think about this approach is that for each subject, a unique threshold is set at the value of their unimpaired condition and any balance score given that is ‘worse’ than that is considered ‘impaired’. In that sense, this method of impairment detection uses a subject-specific threshold for each subject. We considered an impairment detected correctly by a balance metric if for a given subject the impaired condition score was rated ‘worse’ than the same subject's unimpaired score. This use of a subject-specific threshold is only feasible in this study because of our experimentally imposed artificial impairments. In a clinical setting, this would require comparing a patient's balance metric score to that patient's healthy baseline measurement.

#### *Post hoc* stride detection

For all balance metrics evaluated using stride detection, we used a *post hoc* detection of heel strike to identify the start of a stride. We used the center of pressure of the person on the treadmill to detect each heel strike. When the subject's foot contacted the treadmill at the end of the swing phase, the center of pressure measured by the treadmill moved rapidly. We first low-pass filtered the center of pressure position signal with a fourth-order, zero-lag Butterworth filter with a 6 Hz cutoff frequency. We detected the time point when the fore-aft center of pressure velocity rose above 0 ms^−1^ in the laboratory frame and defined this as a potential heel strike. We also detected the time point when the medial-lateral center of pressure velocity rose above 0.7 ms^−1^ and defined this as a potential heel strike. During some strides, both thresholds were triggered as potential heel strike events, while in others, only one threshold was triggered. This variation depended on where the swing foot was placed relative to the stance foot. When both thresholds were triggered within a predefined short time window, the first detected heel strike event was selected as the valid detection. The size of the predefined time window differed depending on the trial type. For perturbation trials, a 150 ms window was applied to account for the rapid step succession. For steady-state walking, a 200 ms window was used as steps occur at a regular cadence. We included additional logic to remove ‘stutter steps’ and rapid changes in center of pressure that were not related to a proper stride but instead caused by recovery motion. We considered a stutter step to be when a subject lifted off a foot but did not place it in front of the contralateral leg on the treadmill. We tracked the foot's position and established the alternating pattern of foot placement starting 7 s before each perturbation onset. We excluded potential heel strike events if the same foot from the previous heel strike remained in front and required foot markers to be less than 0.2 m above the treadmill surface. The logic to remove stutter steps and rapid changes in center of pressure underfoot was not triggered during any steady-state walking periods and was triggered during 5.2% of perturbation recovery trials in the 2 s following a perturbation onset. Of these recoveries triggering the stutter step logic, 94% occurred during high magnitude (15% bodyweight) perturbations, and 82% occurred during medial-lateral perturbations. These results suggest higher magnitude and medial-lateral perturbations elicit especially strong stepping responses to perturbations.

#### Data preprocessing

Marker data were filtered using a fourth-order, zero-lag Butterworth filter with a cutoff frequency ranging from 8 to 25 Hz, with the specific frequency chosen as appropriate for each balance metric calculation. The center of mass was estimated as the arithmetic mean of motion capture markers placed on the pelvis and its velocity was computed using the finite difference method (Kwon et al., 2023; Mahaki et al., 2023; Wang and Srinivasan, 2014). The center of mass signals were further filtered with a 25 Hz fourth-order, zero-lag Butterworth low-pass filter.

#### Step-width variability

**Fig. 4.**
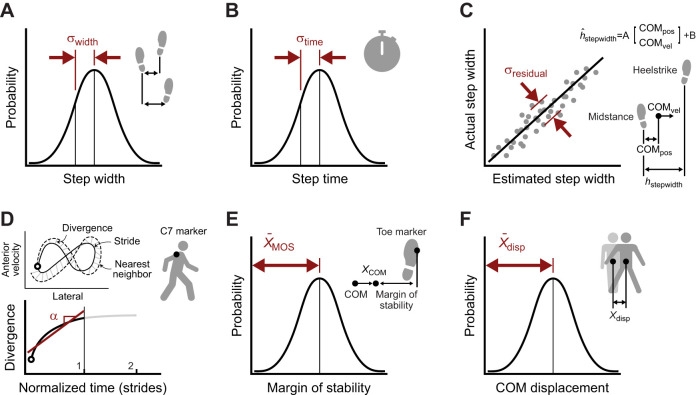
**Balance metrics.** We evaluated (A) step-width variability, (B) step-time variability, (C) lateral foot placement predictability, (D) Lyapunov exponent, (E) lateral margin of stability and (F) center of mass (COM) displacement after a perturbation. MOS, margin of stability; COM_pos_, center of mass position; COM_vel_, center of mass velocity; disp, displacement.

Step-width variability was calculated by first determining the step width for a series of steps and then calculating the variability for a specified range of steps (Fig. 4). Step width was calculated by detecting heel strikes using the ground reaction forces of the instrumented treadmill, as described in the ‘*Post hoc* stride detection’ section, and then finding the distances between the heel markers. The medial–lateral distance between the subject's calcaneus markers was calculated for each step. A positive distance was defined as when the feet were not crossed (i.e. left foot was to the left of the right foot at heel strike) and negative step width was defined as when the feet were crossed (i.e. right foot was to the left of the left foot). Step width was calculated for both the steady-state walking trials and the perturbation trials. To estimate step-width variability during only steady-state walking, the unperturbed walking trials were used as well as step data immediately preceding a perturbation from the perturbation trials. These steps preceding a perturbation occurred 18–23 s after the previous perturbation, when the subject had completed their recovery and was once again walking at steady state. To estimate step-width variability during steady-state plus perturbation recovery, step data from steady-state walking trials were included as well as step widths from the seconds immediately after a perturbation (7 s per perturbation). Because step-width variability estimates can be biased by the number of steps included (Desmet et al., 2019; Owings and Grabiner, 2003), the number of steps used to calculate variability was trimmed to the minimum number (570 steps) across subjects and conditions. This blocks against systematic bias by including different numbers of steps in the variability calculations. Finally, the variability was calculated for each subject and balance condition. We considered higher variability to indicate worse balance.

#### Step-time variability

Step-time variability was calculated the same way as step-width variability except instead of calculating the distance between heel markers at each heel strike, the time elapsed since the previous heel strike was used (Fig. 4). To ensure asymmetry did not bias the estimates of variability, steps landing on the left foot and steps landing on the right foot were stored in separate distributions, both distributions were made zero-mean and then the two distributions were combined before calculating the standard deviation. We used the same number of steps (570) to calculate step-time variability across subjects in all conditions to avoid systematic bias by including a different number of steps in the variance calculations. We considered higher variability to indicate worse balance.

#### Foot placement predictability

Foot placement predictability was calculated by comparing expected foot placement to true foot placement (Fig. 4). Expected foot placement was calculated using the center of mass position and velocity at midstance to explain variance in medial-lateral foot placement in the following heel strike, as described with a linear model (Wang and Srinivasan, 2014). A separate model was fit for each subject and condition. The linear model accepted lateral pelvis position relative to the calcaneus marker of the stance foot and lateral pelvis velocity in the laboratory frame at midstance as inputs. The model output was the lateral position of the calcaneus marker on the swing foot relative to the calcaneus marker on the stance foot at heel strike. Foot placement predictability was quantified as the standard deviation of the error of the model's predicted foot strike relative to the true foot strike position (Mahaki et al., 2023). Position of the center of mass and location of heel strike were measured relative to the heel marker of the current stance foot. We acknowledge that while lateral pelvis position was calculated in the calcaneus reference frame, velocity was defined in the laboratory frame. Although this distinction did not affect the results in the mediolateral direction presented in this study owing to the nominal difference in velocity definitions, future work should consider unifying these state definitions for improved clarity and consistency in interpretation.

Only medial–lateral components of position and velocity measurements were used for foot location and center of mass state. As described in the ‘Step-width variability’ subsection, we used the same number of steps (570) to calculate foot placement predictability across subjects in all cases. We considered higher variability to indicate worse balance.

#### Lyapunov exponent

The Lyapunov exponent approximation was implemented using an algorithm, state definition, time-normalization method and hyper parameters from previous work (Bruijn et al., 2009; Dingwell and Cusumano, 2000; Rosenstein et al., 1993). Previous work has described in detail the method for approximating the maximum Lyapunov exponent using experimental gait data (Bruijn et al., 2009; Dingwell and Cusumano, 2000). A brief description of how we implemented this method follows. We first selected the state to be the linear velocity of the C7 marker in three dimensions in the laboratory frame. We then time-normalized the state trajectories by stride so each stride had 100 time-steps. We then converted the time-normalized state trajectory to an embedded state trajectory, *S*(*t*), using the method of time-delayed embedded dimensions:
(1)

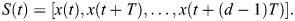


We used four time-delayed states (*d*=5), appended to the original state, resulting in a 15-element embedded state array. Each time-delayed state appended to the original state was delayed by the same incremental delay, *T*, of 10 normalized timesteps. For each point in this embedded state trajectory, the nearest neighbor point was found in other strides and the difference between the trajectories was calculated. The L_2_ norm of the difference between these trajectories was calculated at each subsequent time point and formed a record of the divergence of the two trajectories from the original nearest neighbor point. The natural log was taken for each point of this trajectory. At each timepoint in normalized time the mean across these trajectories was taken to generate a mean divergence trajectory. The maximum Lyapunov exponent was found by fitting a linear model to this mean divergence trajectory using data from the first stride, where the slope approximated the maximum Lyapunov exponent (Fig. 4). Only short-term data from the first stride were used to calculate an estimate of the Lyapunov exponent because this method is thought to have a stronger correlation to fall history than calculations using data from more than one stride (Toebes et al., 2012).

The number of strides used to estimate the Lyapunov exponent influences the magnitude of the exponent, so each estimation was limited to 144 strides for sufficient sensitivity to changes while remaining computationally tractable (Bruijn et al., 2009). For steady-state evaluation, we used 144 strides from the unperturbed walking trials preceding and following the perturbation trials. Each of the two unperturbed walking trials was broken into eight sections and nine strides were used from each section. For the steady-state plus perturbation data evaluation, we used nine strides for each of the perturbation trials with the perturbation beginning during the fifth stride included, again totaling 144 strides per subject and condition. We considered a higher Lyapunov exponent to indicate worse balance.

#### Lateral margin of stability

Lateral margin of stability was calculated using a previously established approach (Peebles et al., 2016). The pendulum length was calculated for each stride as the distance between the ankle marker and the center of mass. The extrapolated center of mass was calculated by using the center of mass position at heel strike and adding the center of mass velocity divided by the natural frequency of an equivalent pendulum. The medial-lateral distance between the extrapolated center of mass and the motion capture marker on the fifth metatarsal of the foot at heel strike was taken to be the margin of stability (Fig. 4). We chose to use this implementation of margin of stability because it was most commonly used in previous work (Beltran et al., 2014; Gates et al., 2013; Hurt and Grabiner, 2015; Lencioni et al., 2021; Peebles et al., 2016; Rodrigues et al., 2021; Tisserand et al., 2018). The mean of the margin of stability across many strides was taken as the final outcome measure for each subject and condition. For steady-state walking evaluation, the unperturbed walking trials were used as well as the 7 s preceding a perturbation during the perturbation trials. For steady-state walking and perturbation evaluation, the unperturbed walking trials were used as well as 7 s following a perturbation in the perturbation trials. We used the same number of steps (570) across all subjects and conditions. To be consistent with prior studies, we designated higher lateral margin of stability as indicating lower balance ability.

#### Center of mass displacement

Center of mass displacement as a result of a perturbation is the only balance metric we evaluated that was initially developed for use on perturbation recovery data rather than during steady-state walking. Perturbation-based balance signals have previously been studied (Bruijn et al., 2010, 2022; Fallahtafti et al., 2023; Kwon et al., 2023; Sheehan et al., 2015; Vlutters et al., 2016, 2018) and metrics using these signals are supported by their intuitive rationale that studying an individual's recovery from a mechanical perturbation that challenges their balance will likely reveal information about that individual's balance ability. Inspired by previous work (Kwon et al., 2023), we chose to implement an especially intuitive balance metric measuring how far the center of mass moved sideways following a sideways perturbation. Some slight differences between the perturbations applied in the study that inspired this metric and the perturbations in our study prompted us to slightly modify the calculation in an attempt to retain the intent of the metric. In our study, we looked at the medial–lateral displacement of the center of mass at the point of midstance relative to the position of the center of mass at the previous midstance point (Fig. 4). To ensure consistency and comparability with the previous study (Kwon et al., 2023), the evaluation of this metric on perturbation recovery data was limited to two displacement measurements: one from each of the low-magnitude (7.5% bodyweight) rightward perturbations occurring at midstance of the left leg. Displacement of the center of mass was measured from the position of the center of mass at the onset of the perturbation (midstance of the left leg) to the center of mass position at midstance of the left leg during the next gait cycle. The arithmetic mean of these two displacements was taken as the outcome measure. The results for this calculation appear under the same category of ‘steady-state plus perturbation’ data used for other metrics, but for this metric, no steady-state data were used, only perturbation recovery data, because these most accurately reflect the original description and are most likely to be a meaningful measure of balance (Kwon et al., 2023). For steady-state walking evaluation, the unperturbed walking trials were used as well as the 7 s preceding a perturbation during the perturbation trials. We used the same number of steps across all subjects and conditions (570). We evaluated many variations of this metric using different definitions for displacement magnitude, direction, and timing, and the method described resulted in the highest accuracy for detecting impaired balance. We considered larger displacements to indicate worse balance.

### Statistics

We used bootstrapping to increase the confidence in the results of our study. Bootstrapping is an effective tool for improving estimation accuracy and informing the confidence level appropriate for conclusions drawn from a distribution of data (Chernick, 2011). For this study, this improved the estimates for detection accuracy of impaired balance and provided information on how sensitive each balance metric was to the subset of data used in its calculation. We used 1000 bootstrap samples for all metrics except Lyapunov exponent, which used 100 bootstrap samples owing to the high computational demand. Each bootstrap sample was generated by sampling with replacement from the original data until the bootstrap sample was 70% the size of the original. Each bootstrap sample for the Lyapunov exponent used 99 of the 144 strides originally selected for evaluation and all other metrics used 400 of the original 570 steps originally selected for evaluation. Each bootstrap sample was used to calculate the detection accuracy using the metric, threshold and motion of interest. This produced a distribution of accuracies for each metric, threshold type and option of steady-state or perturbation recovery data used. We used these bootstrapped distributions for all statistics except for evaluation of learning effects.

We used separate statistical analyses to test for the significance of the effect of using subject-specific versus cohort-based thresholds, steady-state versus steady-state plus perturbation recovery data and the effect of the balance metric used. We used a Wilcoxon signed-rank test to determine statistical significance of the subject-specific versus cohort-based threshold. We also used a Wilcoxon signed-rank test to determine statistical significance of using steady-state versus steady-state plus perturbation recovery data. To assess the importance of the metric used, we first ran a Kruskal–Wallis test across the different metrics. We performed a contrast using a Wilcoxon rank sum test to determine if step-width variability, step-time variability and foot placement predictability performed better than the other metrics. We also evaluated whether learning effects were present in metrics with high detection accuracy by calculating the metrics across time, fitting a linear model to the metric values over time, and testing if the change in metric over time was statistically significant using a Wilcoxon signed rank test. Owing to the multiple comparisons done to evaluate the effect of threshold, data used, balance metric performance, and presence of learning effects, we used a Bonferroni correction to adjust the threshold for statistical significance from α=0.05 to α=0.0071 for all tests.

## RESULTS

**Fig. 5.**
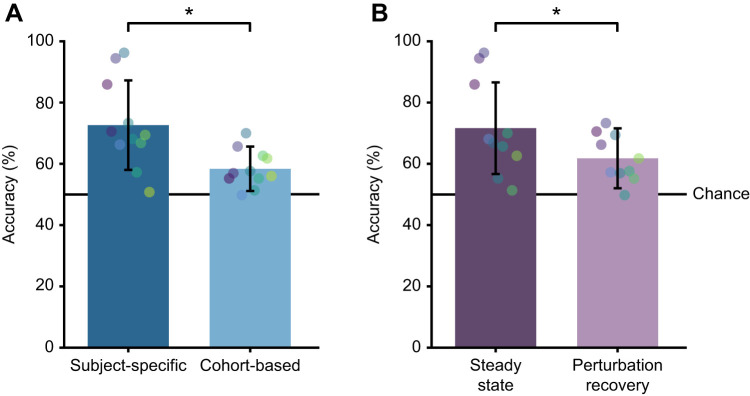
**Comparison of thresholding and perturbation techniques.** (A) Detection accuracy using subject-specific versus cohort-based thresholds. Subject-specific thresholds result in significantly higher detection accuracy (*N*=11 metrics, *P*<1.0e−16). (B) Detection accuracy using steady-state data versus steady-state plus perturbation data. Using data from steady-state walking resulted in significantly higher detection accuracy (*N*=10 metrics, *P*<1.0e−16). Each dot represents one metric's performance, with consistent colors indicating the same metric across bars. Bars are means±s.d. with individual data points.

We found that using a subject-specific threshold rather than a cohort-based threshold improved detection of impaired balance (Fig. 5A, paired Wilcoxon signed-rank test, *P*<1.0e^−16^). When using results from both steady-state and steady-state plus perturbation recovery data for all metrics except center of mass displacement, which used only perturbation recovery data, subject-specific thresholds resulted in a mean detection accuracy of 73±15% whereas cohort-based thresholds had a mean accuracy of only 58±7% (chance was 50%).

We found that using steady-state data resulted in a higher detection accuracy (72±15%) than steady-state plus perturbation recovery data (62±10%) when using subject-specific and cohort-based thresholds and averaging across all metrics except center of mass displacement (Fig. 5B, paired Wilcoxon signed-rank test, *P*<1.0e^−16^). We used these results to inform our final comparison of the balance metrics.

**Fig. 6.**
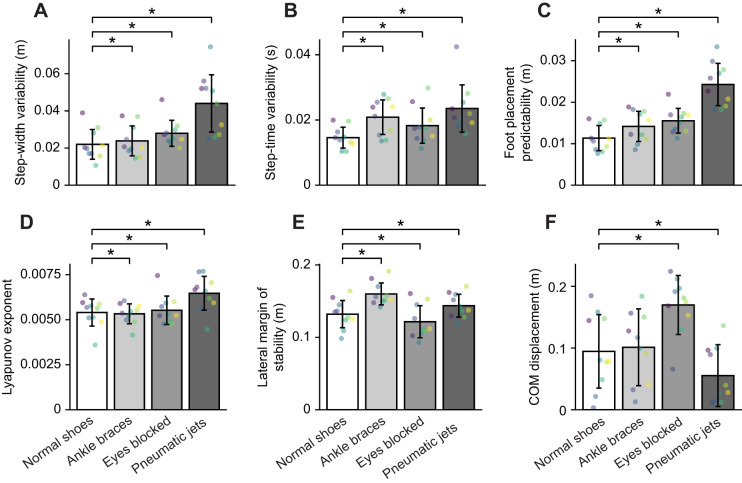
**Numerical scores for each metric across impairment conditions.** (A) step-width variability, (B) step-time variability, (C) lateral foot placement predictability, (D) Lyapunov exponent, (E) lateral margin of stability and (F) COM displacement after a perturbation. All metrics used steady-state data except COM displacement, which used perturbation recovery data. Bars represent means±s.d. of the metric across all subjects (*N*=10). Each dot represents one subject's metric score, calculated as the mean of all bootstrap samples. Dots sharing the same color correspond to the same subject across all bars. *P<0.05.

**Fig. 7.**
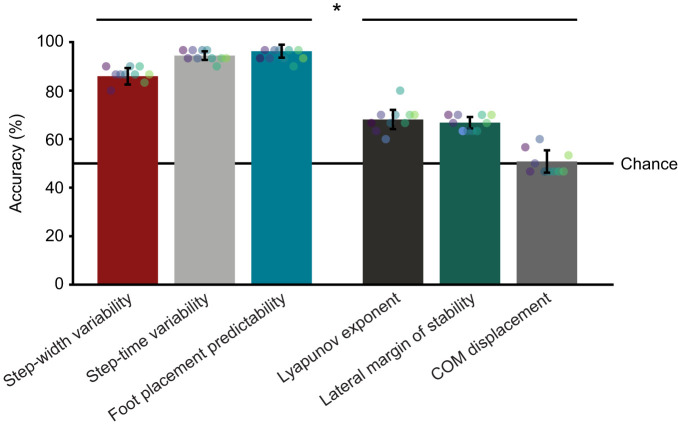
**Comparison of metric accuracies.** All metrics used steady-state data except for center of mass displacement, which used perturbation recovery data. The metrics using information about variability are grouped and the remaining metrics are grouped for statistical analysis, as indicated by asterisk over bars. The metrics using variability have a significantly higher detection accuracy (*P*<1.0e^−16^). Bars are means±s.d. across bootstrap samples for all subjects calculated from 30 comparisons. Each dot represents a subject's average metric score (mean of bootstrap samples), with consistent colors indicating the same subject across bars.

We found that different metrics had a wide range of sensitivity to the balance impairments. We compared impairment detection accuracy across different metrics using subject-specific thresholds. For all metrics except center of mass displacement, we used steady-state walking data; for center of mass displacement, we used perturbation recovery data. We found that the metric used had a significant effect on impairment detection accuracy (Kruskal–Wallis test, *P*<1.0e^−16^). The varying levels of sensitivity each metric had to the different impairments is visually apparent when plotting metric scores averaged across all subjects and grouped by impairment condition (Fig. 6). We also performed analysis where the metrics were normalized by each subject's leg length (Fig. S1A). The results showed no significant differences between the normalized and non-normalized metrics (Fig. 6). Step-width variability (86±3%), step-time variability (94±2%) and foot placement predictability (96±3%), all had significantly higher detection accuracy (≥86%) than the Lyapunov exponent (68±4%), lateral margin of stability (67±2%) and center of mass displacement (51%±5%) (≤68%, Fig. 7, contrast between the two groups performed with unpaired Wilcoxon rank sum test, *P*<1.0e^−16^). Owing to the number of subjects and conditions, 30 comparisons were used to compute the subject-specific threshold accuracy and 40 comparisons were used to calculate cohort-based detection accuracy for each bootstrap sample and on average produced similar detection accuracies to not using bootstrapping (Table S1A).

Plots showing the detection accuracy using all metrics, detection thresholds and steady-state versus steady-state plus perturbation data are shown in Fig. S1B. Plots showing all balance metric scores for individual subjects and subjects grouped by condition are available in Figs S2 and S3. Individual subject scores and group-level scores for step length, step width and anterior margin of stability are also provided in the Figs S4 and S5. Using steady-state data and subject-specific thresholds, anterior margin of stability detected impaired balance with an accuracy of 13%.

Presence of learning was assessed using step-time variability, step-width variability and step width by comparing how values changed from the first 200 steps to the last 200 steps in a condition. Averaged across subjects and conditions, step-time variability was 53% higher in impaired conditions. Averaged across all conditions, it significantly decreased by 10% from the beginning to the end of a condition (−0.18 ms min^−1^, *P*=1.2e^−4^, Wilcoxon signed rank test). Step-width variability was 54% higher in impaired conditions and did not change over time (0.022 mm min^−1^, *P*=0.26, Wilcoxon signed rank test). Step width increased by 30% on average during impaired conditions and significantly decreased by an average of 8.5% from the beginning to the end of a condition (−0.91 mm min^−1^, *P*=4.5e^−5^, Wilcoxon signed rank test).

## DISCUSSION

### Subject-specific versus cohort-based threshold

Using subject-specific thresholds substantially improved the accuracy of impaired balance detection (Fig. 5A). One reason that subject-specific thresholds may outperform cohort-based thresholds is that they control for differences in movement patterns between individuals. The differences between individuals reported by a balance metric may reflect differences in balance ability or differences in movement patterns unrelated to balance. Using subject-specific thresholds will only be effective for detecting real impairments in patient populations if the threshold is set while a subject has relatively unimpaired balance. If the threshold was not set while a subject has healthy balance, subject-specific thresholds could still be useful for informing balance interventions such as training or device assistance; because as long as balance is improving relative to the subject's pre-intervention threshold, the intervention is helping.

It is likely that the value from a given balance metric is made up of a combination of information about balance and artifacts of movement unrelated to balance. One way to improve the understanding of the ratio between these two factors and to determine if subject-specific thresholds are necessary for detection of impaired balance would be to conduct a training study with two groups of older adults: a training group and control group. The first step of this study would be to record baseline values for their subject-specific threshold (e.g. step-time variability). The next step would be to train the training group to improve their balance using a method previously demonstrated to reduce fall rate, such as perturbation training (Pai et al., 2014). The final step would be to measure balance ability after the intervention and follow both groups for several months to record falls. These data could be used to determine if high baseline impairment scores or changes from that baseline explain more of the variance in fall rate. This result would inform whether differences in baseline measurements reflect differences in balance ability and whether subject-specific thresholds are helpful when predicting fall risk in patient populations.

The ultimate goal of balance research is to slow the progression of an impairment or, ideally, improve balance to prevent falls. Comparing balance ability across subjects can be interesting but is not necessary for this goal. Results from this study support the idea that within-subject changes detect changes in balance ability with more accuracy than comparing to other subjects and are likely useful for developing or prescribing balance interventions. For example, within-subject changes would be informative feedback for optimizing balance training or device assistance.

Once validated in a large, longitudinal study in patient populations, subject-specific thresholds could have important implications for clinical practice. Subject-specific thresholds could be established with additional clinic visits using laboratory-based motion capture to record gait prior to impairment onset. These additional visits require more clinic time for both patients and clinicians, which would need to be justified by sufficient benefit to the patient, such as using perturbation training to improve balance before a fall occurs (Pai et al., 2014). Wearable sensors could be used to generate subject-specific thresholds without the burden of additional clinic visits and could also continuously monitor patients for early signs of balance impairment. Use of wearable sensors for these measurements is feasible; however, the required accuracy for a wearable system was not evaluated in this study and would need to be established prior to clinical use.

A cohort-based approach inherently requires larger datasets with a wide range of features to account for inter-subject variability in balance metrics. These datasets would need to include diverse anthropometric characteristics, gait patterns and age ranges to capture the variability across individuals. With sufficient data, it might be possible to develop robust normalization methods or clustering techniques to reduce variability unrelated to balance and determine cohort-based thresholds that are broadly applicable. For instance, anthropometric normalization, such as scaling metrics by leg length or pelvic width, could help account for differences in body dimensions. Advanced machine learning techniques could identify the most informative features for balance assessment while filtering out irrelevant variability. Future research should focus on collecting and analyzing large, diverse datasets to explore these possibilities.

### Steady-state versus steady-state plus perturbation data

Using data from steady-state walking resulted in higher detection accuracy of artificially impaired balance relative to using steady-state plus perturbation recovery data (Fig. 5B). While we initially hypothesized that perturbation recovery data would provide additional information about an individual's balance ability, this finding might be influenced by the types of impairments applied, the number and type of perturbations included, and the specific metrics evaluated.

Perturbations may be useful for detecting some but not all forms of balance impairment. The impairments in this study affected both steady-state walking and perturbation recovery motion. Some impairments may affect perturbation recovery more than steady-state walking and for those impairments, perturbations would likely be helpful for identifying impaired balance. Future studies should examine which patient conditions could be more accurately detected using perturbation recovery motion.

It is possible that a greater number of perturbations are needed to improve detection accuracy for these metrics. Calculating an accurate estimate of some measures of gait requires numerous walking cycles. For example, step-width variability requires ≥400 steps to produce an accurate estimate (Desmet et al., 2019; Owings and Grabiner, 2003). The trials in this study contain enough data for this calculation during steady-state walking, but there are far fewer steps from perturbation recovery. Recovery motion is highly variable, so including a small number of recovery steps alongside steady-state walking data may have a dominant effect on outcomes such as variability. In order to induce enough recovery steps for an accurate calculation of variability during recovery, it is likely that many more perturbations would be needed.

We expected center of mass displacement to have high detection accuracy when using perturbation data because it was designed to assess recovery motion, but it had no better accuracy than random chance. It is possible that this measure is less sensitive to balance than other measures. Another reason for the low accuracy could be the number of perturbation recoveries used. The study that inspired this metric used five perturbations per subject and condition (Kwon et al., 2023, which focused exclusively on rightward perturbations with a small perturbation magnitude. To maintain consistency and comparability with this study, we restricted our analysis to the two perturbations per subject and condition that met these criteria. For each perturbation, a single stride was analyzed using the medial–lateral displacement of the pelvis beginning at the onset of the perturbation. This metric uses far less data than other metrics. For example, step-time variability uses 285 strides (570 steps) per calculation compared with the two strides used in the center of mass displacement. Collecting more perturbation recoveries may improve the accuracy of metrics designed to measure balance ability using recovery motion.

The type of perturbation used may also have influenced the accuracy with which the impairments were detected. It is possible that a different onset time in the gait cycle (e.g. heel strike), type of mechanical disturbance (e.g. trip or slip) or modality of disturbance (e.g. galvanic vestibular stimulation) may have made the balance impairments more salient during perturbation recovery. Future studies may find that different types of perturbations increase the accuracy of detecting impaired balance and that specific perturbations are needed to identify specific impairments.

Perturbation recovery metrics have the potential to be useful measures of balance but face a major challenge: they will have access to less data than steady-state walking measures for the same amount of time with a subject. This is because each perturbation only requires a few steps to recover from and the time between perturbations adds time to the session, meaning that measures using perturbation recovery motion will have access to far less data than a metric using steady-state data from the same amount of subject time. Balance metrics designed to extract information from recovery data could be effective, but they will need to be extremely efficient in extracting information from recovery data to outperform the steady-state measures used on much more data.

### Metrics

Step-width variability, step-time variability and foot placement predictability all performed well when using steady-state data and subject-specific thresholds. One commonality of these high-accuracy metrics is that they all describe some type of variability in gait. Step-width variability and step-time variability simply capture the variability in a series of steps, while foot placement predictability describes the variability of medial–lateral foot placement after controlling for some of the variability that is explained by the motion of the center of mass. The high detection accuracy of the impairments in this study by all the metrics sensitive to variability suggests that variability may be an effective way to detect impaired balance.

Each impairment affected balance through a different mechanism and all of these mechanisms increased variability, further supporting the idea that variability and balance are related. The ankle braces likely restricted the ability to make small adjustments in push-off, leading to more control effort through foot placement (Vlutters et al., 2018). The eye-blocking masks likely impaired detection of slight changes in body state, resulting in greater deviations with each step. The pneumatic jets directly injected noise into the placement of the foot, leading to less precise corrections of body state and larger future corrections. It is a common misconception that decreased variability could indicate impaired balance. This claim is based on 3 of 500 subjects in a study in which only a handful of strides were recorded (Brach et al., 2005). The majority of previous work indicates that gait impairments and fall risk are associated with higher variability, although many of these studies are also limited because too few strides are used to accurately estimate variability for each subject (Ayoubi et al., 2014; Brach et al., 2007, 2005; Hausdorff et al., 2001; Heitmann et al., 1989; Maki, 1997; Moe-Nilssen and Helbostad, 2005; Owings and Grabiner, 2004; Svoboda et al., 2017). We believe the high detection accuracy of these variability-based measures across the three distinct artificial impairments further supports the link between variability and balance ability.

Step-time variability, step-width variability and foot placement predictability all measure variability using slightly different measurements, which have a wide range of ease with which they can be recorded. Step-time variability is the easiest to capture because it could be easily measured with wearable sensors (Del Din et al., 2016, 201; Mellone et al., 2012; Wu, 2022; Wu et al., 2021). Step-width variability is the next easiest because it could likely be measured with IMUs but would require a sophisticated sensor fusion algorithm to estimate the kinematic information accurately using IMUs (Teufl et al., 2019; Zhang et al., 2018). Foot placement predictability requires estimating the center of mass position and velocity relative to the feet, which was performed using optical motion capture in this experiment and would be more challenging to measure accurately using IMUs (Leestma et al., 2024; Wang et al., 2025; Zhang et al., 2018). Using steady-state data and subject-specific thresholds, foot placement predictability had the highest detection accuracy of all the metrics (96%), followed by step-time variability (94%) and step-width variability (86%). Foot placement predictability may have had higher detection accuracy than step-width variability because using information about pelvis motion may control for variability unrelated to impaired balance and increase the signal-to-noise ratio. Step-time variability produced the most consistent results across bootstrap samples (Fig. 7 and Fig. S1A). Selecting the most suitable metric for a study will depend on study constraints such as the feasibility of collecting pelvis state measurements, the need for precision in the measurement, and importance of overall accuracy.

The low accuracy of the Lyapunov exponent as a measure of impaired balance in this study may be a result of its reliance on an incomplete system state. The implementation used in this paper used the velocity of the C7 marker (Bruijn et al., 2009). While motion of the torso is likely related to human balance, it may be that a different system definition would more accurately detect changes in balance. For example, foot placement is a critical part of balance and it is possible that supplementing the torso velocity state with information about the subject's feet would improve detection accuracy. The state used in a Lyapunov exponent approach will always be incomplete, given the complexity of the human body system, which may indicate a fundamental limitation to this kind of approach to measuring balance. Future work is needed to establish a consensus on the state used with the Lyapunov exponent approach to measure balance, but given the poor detection accuracy in this study, additional computational requirements of this method, and potentially fundamental limitations in using an incomplete system state, other metrics seem more promising.

Margin of stability is based on a restrictive model of human movement that may inhibit its ability to detect impaired balance. People with impaired balance may be closer to a state where they may fall, and they may also try to compensate for their impaired balance by keeping themselves far from a state resulting in a fall. Unfortunately, margin of stability may be sensitive to both the impairment and the compensatory behavior. This sensitivity to compensatory behavior has been suggested to explain why in previous studies lateral margin of stability indicated higher stability for conditions in which people were more likely to fall (Gates et al., 2013; Rethwilm et al., 2021; Rodrigues et al., 2021; Tisserand et al., 2018). Detecting both the impairment and compensation will inherently confound the measurement since compensation may shift the metric in the opposite direction as impaired balance and different impairments could result in different compensation strategies. The results in our study demonstrate this trend with ankle braces and pneumatic jets conditions increasing lateral margin of stability and the eyes-blocked condition decreasing lateral margin of stability (Fig. 6). Another limitation of the margin of stability is that it is calculated using the mean of many steps, not capturing the step-to-step adjustments that are used to maintain balance. Instead, capturing fluctuations between steps by using a variability-based calculation may improve detection accuracy. Lateral margin of stability had low detection accuracy in this experiment when other, more easily estimated, metrics were able to detect the impairments, suggesting that other metrics seem more promising.

In addition to evaluating lateral margin of stability, we also evaluated anterior margin of stability for completeness. Previous work has shown that anterior margin of stability has a positive correlation with gait impairment and fall risk (Peebles et al., 2016). However, we found the opposite in our study. Using this convention had very low detection accuracy (13%, Fig. S5A,B). This effect is likely caused by subjects taking shorter steps when impaired (Figs S4,S5), suggesting the anterior margin of stability was detecting a compensatory behavior in subjects taking shorter steps rather than a change in balance ability. The positive correlation between anterior margin of stability and impairment found in prior work was likely caused by people with impairments walking more slowly, which increased their anterior margin of stability. Inconsistent compensation in walking speed, step length, and step width across populations will make it difficult to implement margin of stability as an indicator of balance ability.

Center of mass displacement did not accurately detect impaired balance. We calculated center of mass displacement as a measure of balance using a single stride for two low-magnitude, rightward perturbations. Each stride began at the onset of the perturbation at midstance. We expected that a larger lateral deviation from the beginning of this stride to the end of the stride during recovery would reflect impaired balance. Unfortunately, this measurement did not accurately detect impaired balance in this study. One possible cause of the low detection accuracy is the heterogeneous effect on center of mass displacement across the impairment conditions. For example, during the eyes-blocked condition, subjects would not have been able to sense how far they moved laterally and would not have been able to recover from a large displacement, whereas in the pneumatic jets condition subjects were able to minimize lateral displacement even more effectively than in the normal shoes condition, perhaps due to a wider step width (Fig. S5C,D). This result suggests center of mass displacement following a perturbation is sensitive to aspects of an impairment not directly related to balance. We also considered using the maximum perturbation magnitude a subject could withstand without falling (while walking in a fall-preventing safety harness) as a perturbation-based measure of balance. The physical space needed for recovering from large perturbations is too large for a treadmill-based system, so we did not pursue this approach. Perturbation-based balance metrics still have great potential and may achieve better sensitivity by incorporating additional state information, such as angular momentum, or evaluating alternate features of the recovery, such as time to return to steady state.

The center of mass displacement evaluated on steady-state data was calculated for completeness and was not expected to accurately detect impaired balance. The fact that this metric had an accuracy nearly equivalent to chance using steady-state data gives us additional confidence in the overall evaluation approach. Additionally, pelvis motion was restricted during steady-state walking prior to a perturbation by the small haptic boundary used for the eyes-blocked condition; this resulted in systematically reduced center of mass displacement during steady-state walking in the eyes-blocked condition compared with the normal shoes condition (Fig. S2F).

Different metrics capture distinct aspects of human movement and are sensitive to certain types of impairments while being less sensitive to others (Fig. 6). Our current single metric may not be sufficiently sensitive to all types of impairments. Future research could explore a tailored approach where metrics are weighted or combined based on the specific impairment being assessed. Additionally, if the type of impairment is unknown in the early stages, developing a more sensitive and multifaceted metric capable of detecting a wide range of impairments would be essential.

Beyond human movement, our findings have broader biological implications for understanding fundamental principles of balance control across species, from bipedal to multilegged animals and even to flying and swimming organisms (Alexander, 2003; Biewener and Daley, 2007; Dickinson et al., 2000; Fish and Lauder, 2006). This is because the underlying balance mechanisms, such as sensorimotor integration, feedback control and adaptive gait strategies, are conserved across species (Dickinson et al., 2000). Variability in movement, often considered as a marker of neuromotor control, plays a crucial role in adaptability and robustness across organisms (Biewener and Daley, 2007; Stergiou and Decker, 2011). For example, insects like cockroaches exhibit remarkable variability in limb movements to navigate uneven terrain, demonstrating how adaptability enhances stability in dynamic environments (Jindrich and Full, 2002). Foot placement predictability is critical not only for human balance but also for animals that rely on precise limb positioning for stability, for example, birds adjust their foot placement with precision during perching and landing to maintain balance (Dakin et al., 2016) and quadrupeds such as goats navigating rocky cliffs depend on accurate foot placement to avoid falls (Birn-Jeffery and Daley, 2012). In contrast, the margin of stability primarily reflects average movement patterns and may overlook cycle-by-cycle adaptations crucial for closed-loop control. For instance, cats exhibit rapid adjustments in limb kinematics during unexpected perturbations, showing the importance of dynamic real-time adaptations over averaged measures (Macpherson and Fung, 1999). Similarly, the Lyapunov exponent, although it reflects the resilience of movement patterns to small perturbations, is often less interpretable in relation to specific task demands and neuromuscular control strategies in animals. These insights reveal the universality of balance control mechanisms and present the potential for cross-species applications in robotics, rehabilitation and comparative biology.

#### Artificial impairments

The effects from the artificial impairments were more subtle than we had expected and more subtle than some patient populations at a high risk of falling. Previous studies have found that step-time variability was about twice as large in older adults who fell compared with older adults who did not fall in a 1-year prospective study (Hausdorff et al., 2001). While our artificial impairments increased step-time variability by about 53% on average. The smaller effect of the artificial impairments may have contributed to the low accuracy of cohort-based thresholds and suggests that subject-specific thresholds may be helpful for detecting more subtle impairments in balance ability.

Learning effects are an important consideration when using artificial impairments, and while learning effects are likely to be present in this study, they are not strong enough to meaningfully confound our results. The increase in variability in the impaired conditions may be due in part to motor learning or impaired balance ability. When subjects experience a new walking condition, they initially undergo periods of learning and adaptation (Choi and Bastian, 2007). Variability is increased during motor learning, perhaps due to intentional exploration as part of the learning process (Abram et al., 2022). Motor learning has been shown to be nearly complete after 12 min of adaptation in a treadmill walking task with similar difficulty to the conditions in our study (Finley et al., 2013), suggesting that subjects had likely finished adapting by the end of an impairment condition in our study. Between the first steady-state walking trial and the final steady-state walking trial of each impairment condition, step-time variability decreased by 10%, step-width variability did not significantly change and step width decreased by 8.5%. This change over time suggests that some adaptation is occurring during each condition. However, the total change in step-time variability and step width (−10% and −8.5%, respectively) is much less than the average increase caused by impairments (53% and 30%, respectively). Since motor adaptation was likely to be complete by the end of the condition, most of the effect observed in the conditions was probably from impaired balance and not motor learning. Additionally, during motor learning it is likely that subjects had reduced balance ability because they were not familiar with how to move in the novel condition. While motor learning likely influenced the results of this study, balance was probably impaired enough for the data in this study to inform how to measure balance ability.

One limitation of this study is that the population used to study balance was healthy and we used artificial impairments. This enabled well-controlled comparison of how an individual moved with and without impaired balance, but also relies on the assertion that the impairments we imposed on subjects affected balance in a meaningful way. These artificial impairments were inspired by real impairments that lead to increased risk of falls, but they were not validated to represent any particular patient population. The mechanisms causing increased fall risk in some patient populations such as cognitive impairment may be difficult to represent with artificial impairments and may be most effectively studied in those populations rather than with artificial impairments. It is worth noting that the metrics with the highest detection accuracy rely on information about gait variability and the pneumatic jets condition directly injected variability into foot placement. This likely increased the detection accuracy of step-width variability and foot placement predictability for that impairment. Nonetheless, step-time variability accurately detected the pneumatic jets condition, suggesting variability in general is a useful measure of balance. Additionally, some aspects of the impairments may have resulted in a motion artifact not related to balance ability, but instead related to how the artificial impairments were implemented. For example, in the eyes-blocked condition, pelvis motion was restricted during steady-state walking prior to perturbations, which may influence how subjects chose to move, independent of the changes in movement related to balance. Despite the limitations associated with implementing artificial impairments to study balance, the findings in this paper are promising. These results should be validated in a study repeatedly measuring balance metrics over time in patient populations at elevated risk of falling and correlate changes in the metrics with changes in fall rate.

### Conclusions

In this study we used artificial impairments to study balance. We found that using subject-specific thresholds resulted in significantly more consistent detection of impaired balance than cohort-based thresholds. We also found that step-width variability, step-time variability and foot placement predictability were all significantly better at detecting impaired balance than other metrics when using steady-state data. Adding a limited amount of perturbation recovery data to steady-state data did not improve the metrics' ability to detect impaired balance. While perturbation recovery data may facilitate impaired balance detection for conditions that inhibit recovery motion, the current analysis suggests that additional or alternative metrics or more perturbation data may be required to detect subtle changes in the ability to recover from large perturbations.

Future work may build on this study using observational studies, longitudinal laboratory-based studies and studies using balance training interventions. All three of these study types could use the increased accuracy of the subject-specific thresholds combined with measurements of gait variability. An observational study design could use cameras in care facilities or wearable devices to monitor gait in residents and to determine whether changes in gait (such as increased variability) correlate with increased risk of falling. These observational studies could control for the effects of various environments as well as behavioral predictors of falling such as type and amount of movement over time. Laboratory-based experiments could study balance in large cohorts of fallers and non-fallers by periodically recording gait over several years and following up regularly to check whether the subjects had fallen. These periodic data collections would enable researchers to establish whether an increase in gait variability correlates with fall risk. An intervention study could assess balance prior to and following a balance training intervention known to reduce fall rates such as perturbation training (Pai et al., 2014). If gait variability does not decrease as a result of training, it could indicate that different metrics are needed to assess the ability to recover from large disturbances and associated fall risk. If gait variability proves to be an effective means of assessing fall risk associated with balance ability, it could be used to improve training interventions and devices intended to prevent falls.

## Supplementary Material

10.1242/jexbio.249339_sup1 Supplementary information
