# Supplementary material for: Detecting artificially impaired balance in human locomotion: metrics, perturbation effects and detection thresholds
Source: J Exp Biol. 2025 May 22;228(10):jeb249339. doi: 10.1242/jeb.249339 (PMC12148027; doi:10.1242/jeb.249339)
Supplement: Supplementary information [file jexbio-228-249339-s1.pdf]

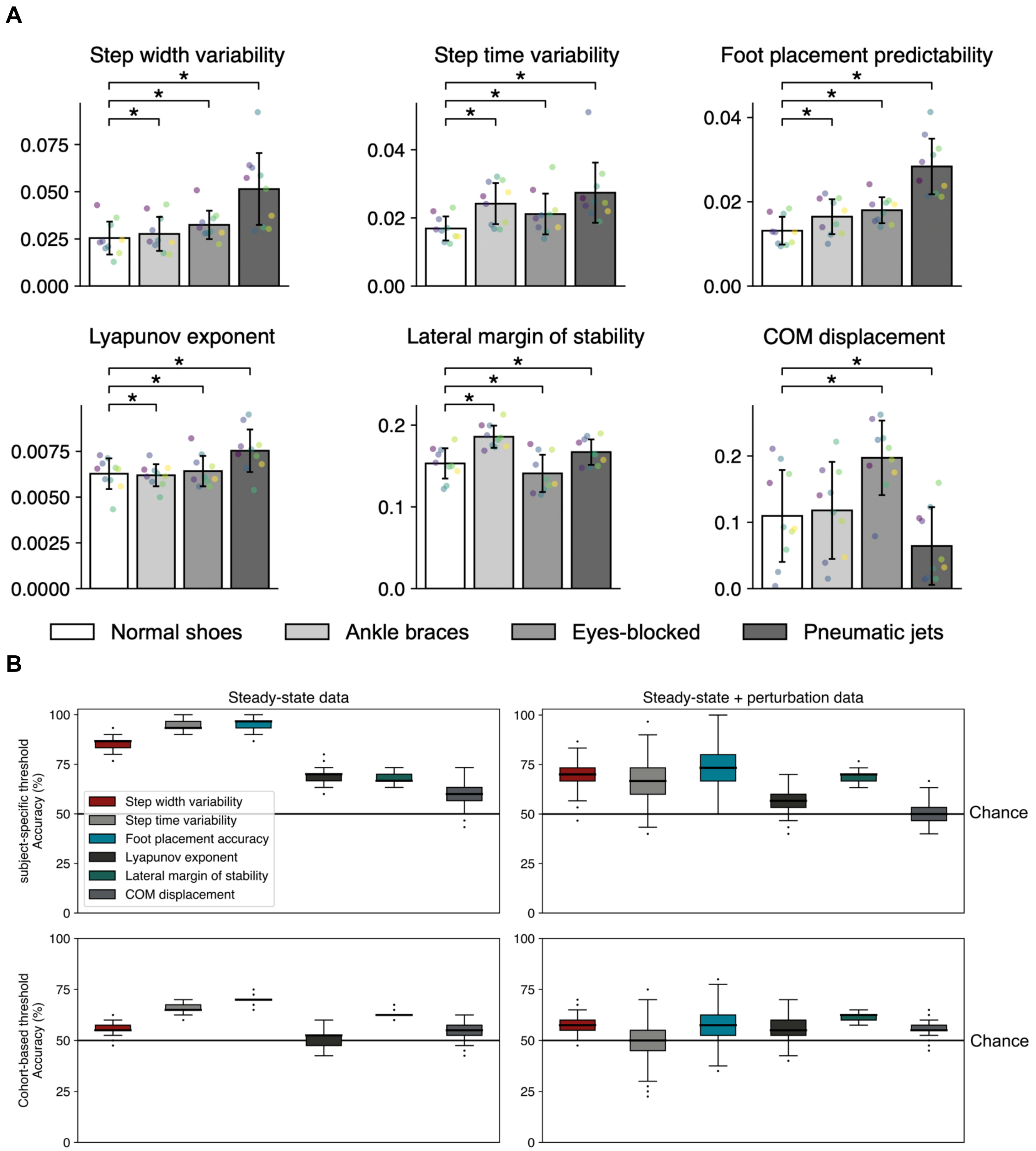

Journal of Experimental Biology • Supplementary information

Fig. S1. A Numerical scores for each metric normalized by participants' leg length across impairment conditions. All metrics used steady-state data except COM displacement, which used perturbation recovery data. Bar height represents mean value of the normalized metric across all 10 subjects and error bars represent standard deviation of the normalized metric score across all subjects. Each dot represents one subject's performance with each unique color corresponding to the same subject. Each subject's metric score was calculated as the mean of all bootstrap samples normalized by leg length. *$p<0.05$. Fig. S1B Plots showing the median and inter-quartile accuracy of detecting balance impairments for each subject. Median is shown with thick black horizontal line within each box. Each box represents the spread of data produced by bootstrapping the original data. All metrics used 1000 bootstrap samples except for Lyapunov exponent, which was limited to 100 bootstrap samples due to computational limitations. The top row of plots uses subject-specific thresholds to detect artificial impairments, and the bottom row uses cohort-based thresholds. The left column uses steady-state data, and the right column uses steady-state plus perturbation data for all metrics except center of mass (COM) displacement, which uses perturbation recovery data only.

Journal of Experimental Biology • Supplementary information

Metric scores for individual subjects using steady-state walking data

A

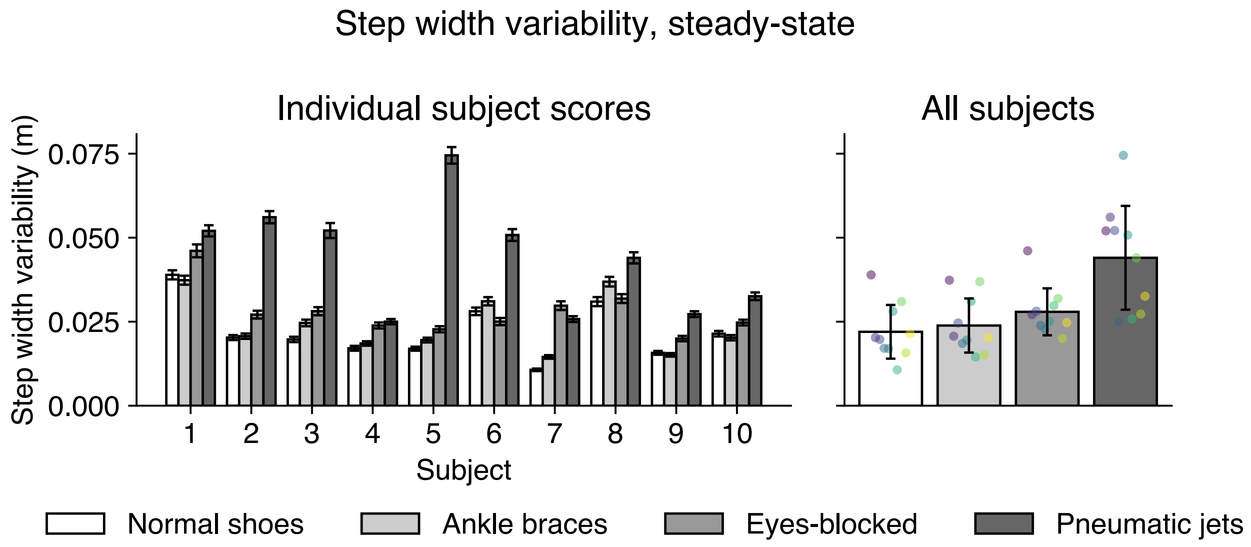

B

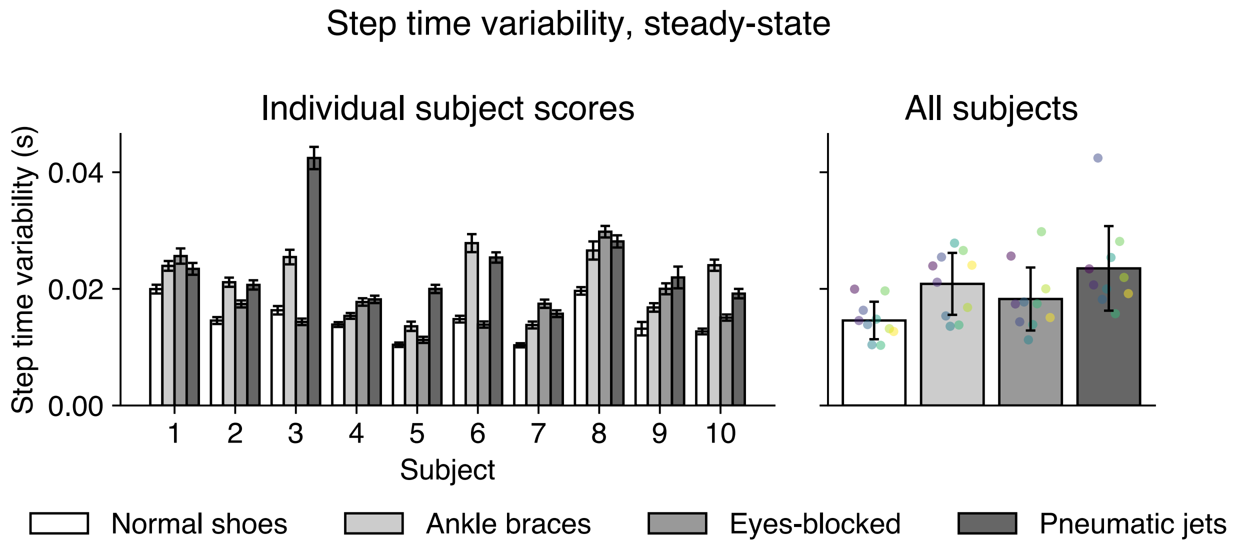

C

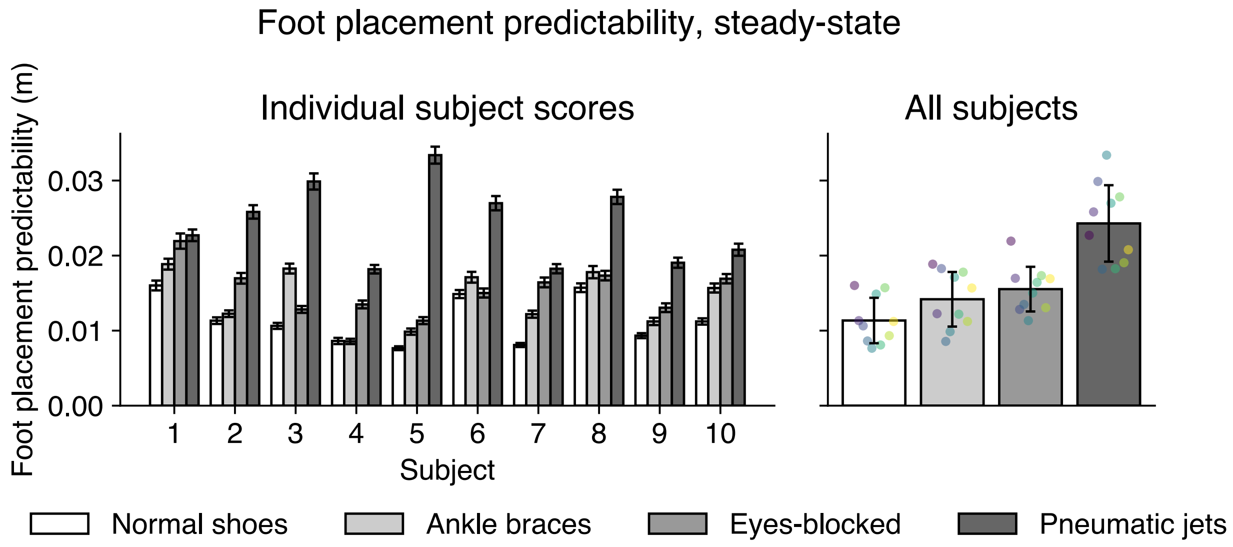

D

Journal of Experimental Biology • Supplementary information

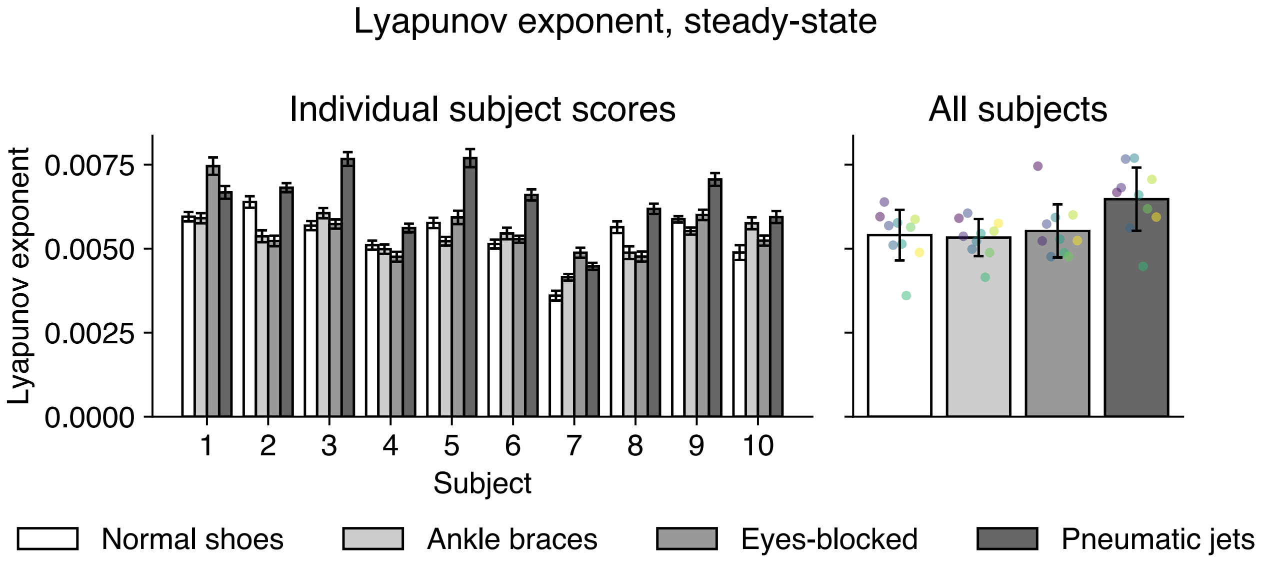
Lyapunov exponent, steady-state
Individual subject scores
All subjects
Lyapunov exponent
0.0075
0.0050
0.0025
0.0000
1 2 3 4 5 6 7 8 9 10
Subject
Normal shoes
Ankle braces
Eyes-blocked
Pneumatic jets

E

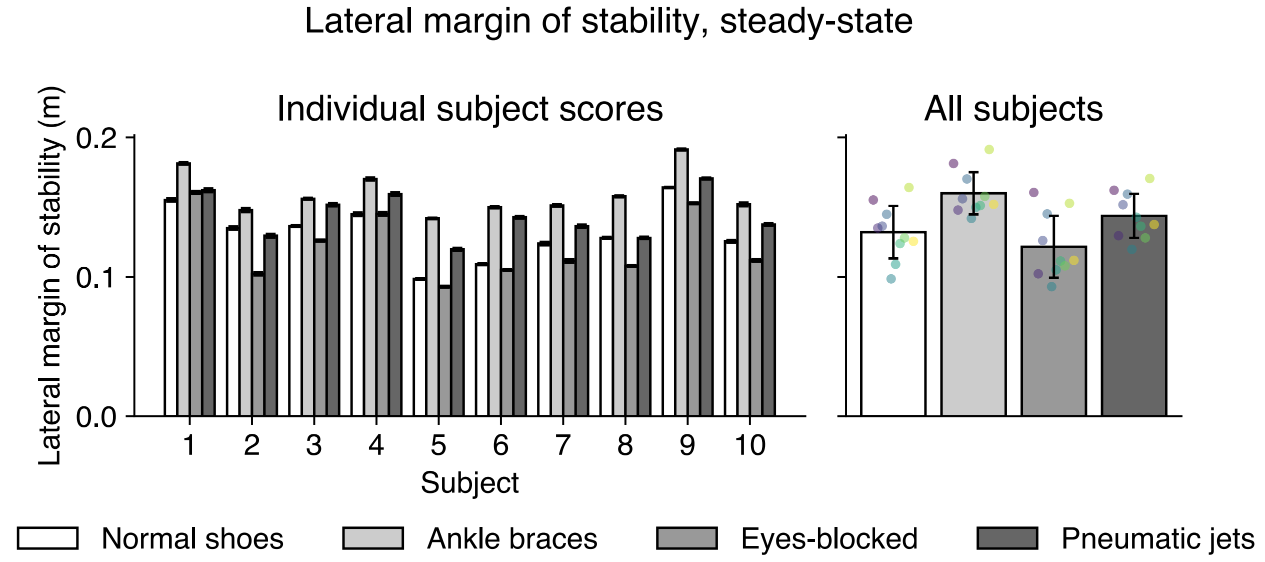
Lateral margin of stability, steady-state
Individual subject scores
All subjects
Lateral margin of stability (m)
0.2
0.1
0.0
1 2 3 4 5 6 7 8 9 10
Subject
Normal shoes
Ankle braces
Eyes-blocked
Pneumatic jets

F

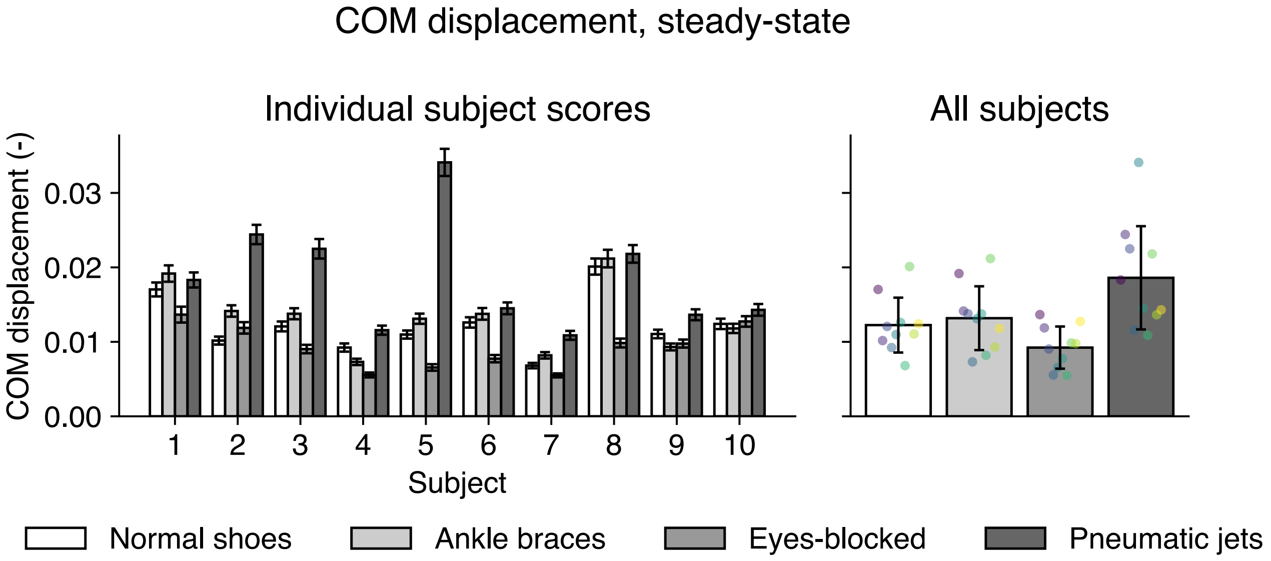
COM displacement, steady-state
Individual subject scores
All subjects
COM displacement (-)
0.03
0.02
0.01
0.00
1 2 3 4 5 6 7 8 9 10
Subject
Normal shoes
Ankle braces
Eyes-blocked
Pneumatic jets

Fig. S2. Plots showing scores for each subject across all four artificial impairment conditions using steady-state walking data. Plots in the left columns show individual scores and plots in the right column show scores averaged across 10 subjects, grouped by impairment condition. Left scores are averaged over 1000 bootstrap samples for all metrics except for Lyapunov exponent, which uses 100 bootstrap samples. Error bars represent standard deviation of the distribution across all bootstrap samples. Each dot in right panels represent mean metric value for a subject, with consistent colors indicating the same subject across plots. Fig. S2A shows step width variability, Fig. S2B shows step time variability, Fig S2C shows foot placement accuracy, Fig. S2D shows Lyapunov exponent, Fig. S2E shows lateral margin of stability, and Fig. S2F shows center of mass (COM) displacement.

Metric scores for individual subjects using steady-state plus perturbation walking data

A

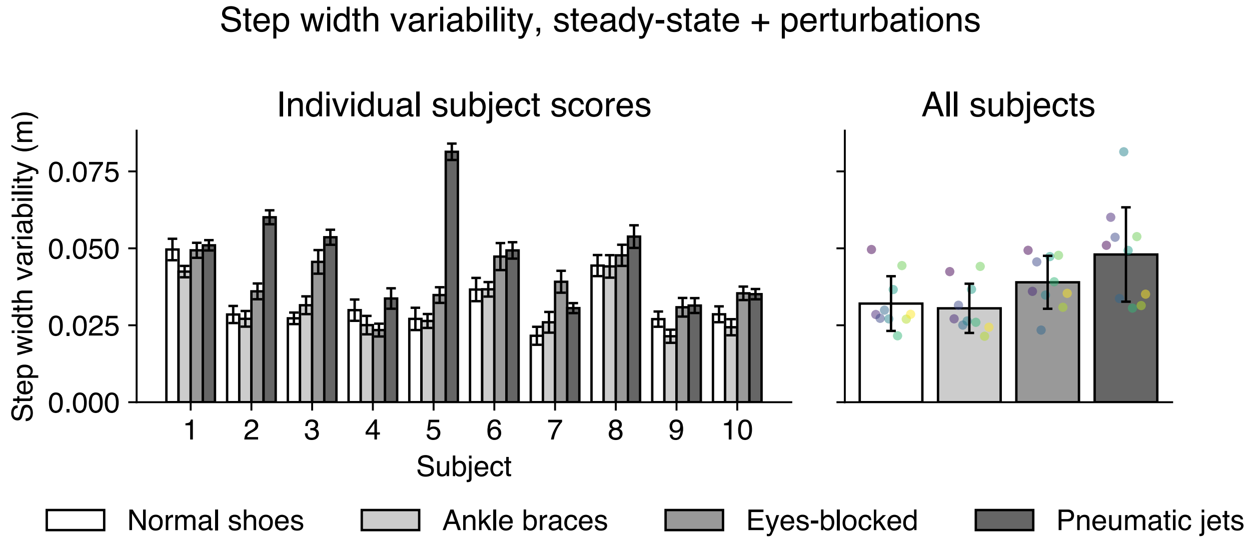

B

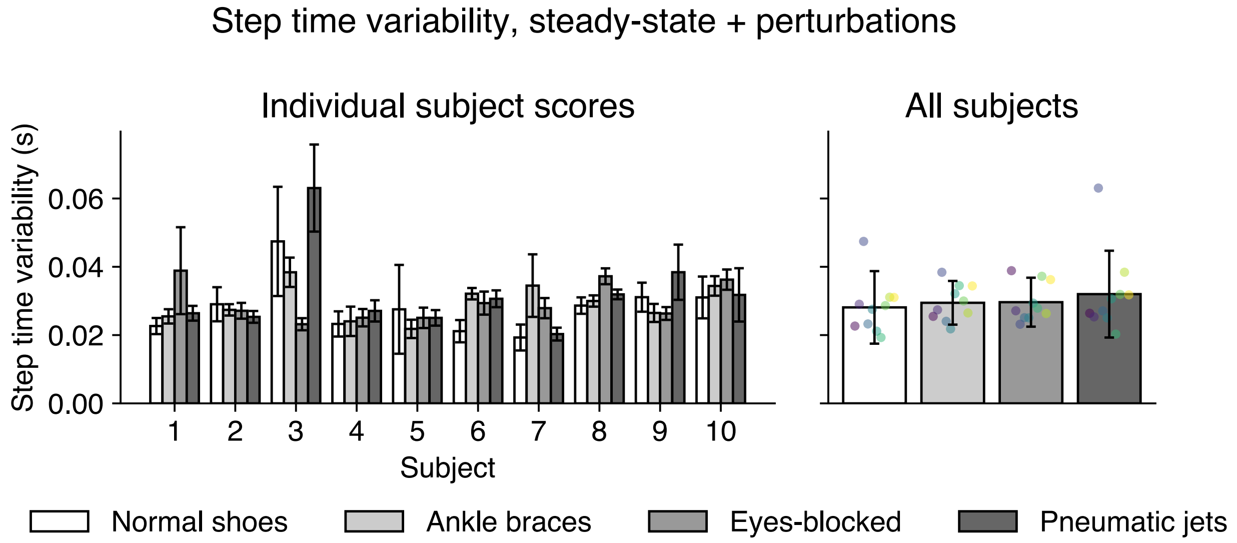

C

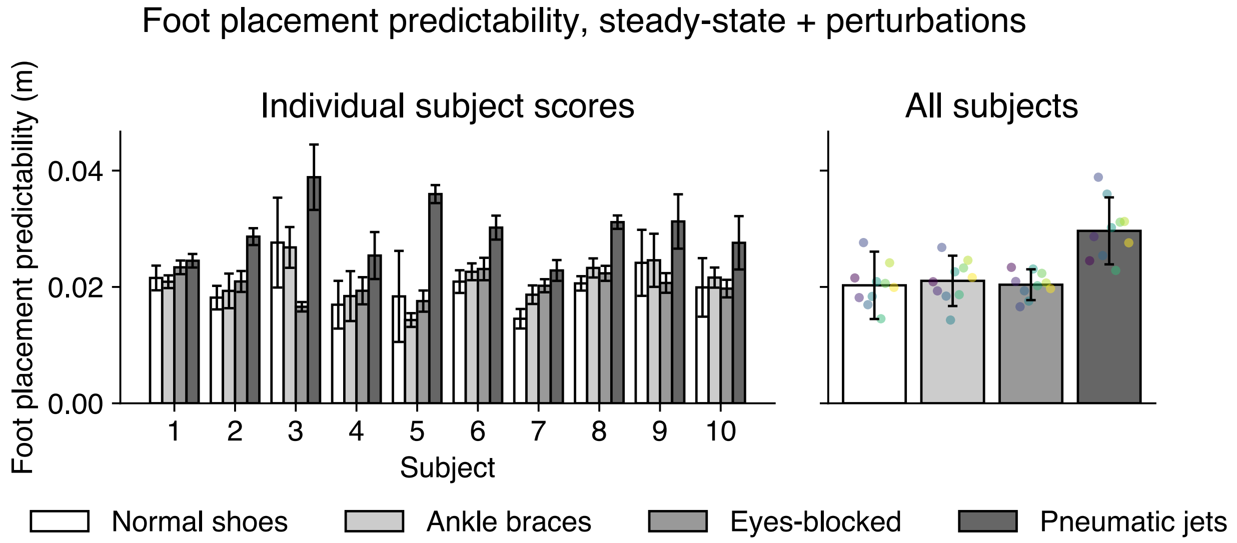

D

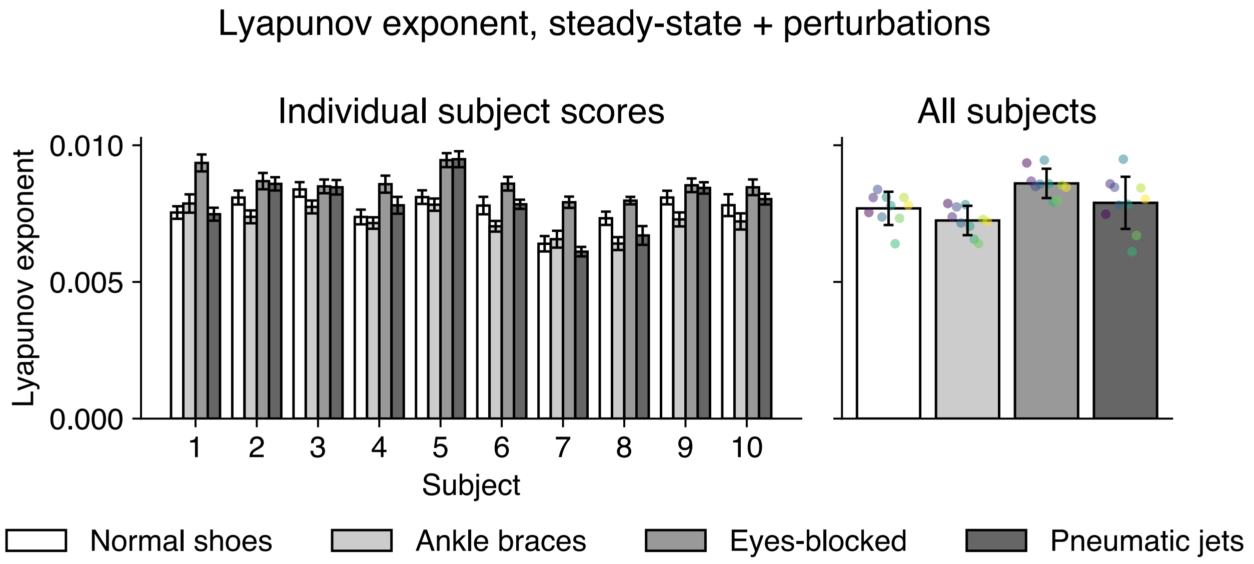

Lyapunov exponent, steady-state + perturbations
Individual subject scores
All subjects
Lyapunov exponent
0.010
0.005
0.000
1
2
3
4
5
6
7
8
9
10
Subject
Normal shoes
Ankle braces
Eyes-blocked
Pneumatic jets

E

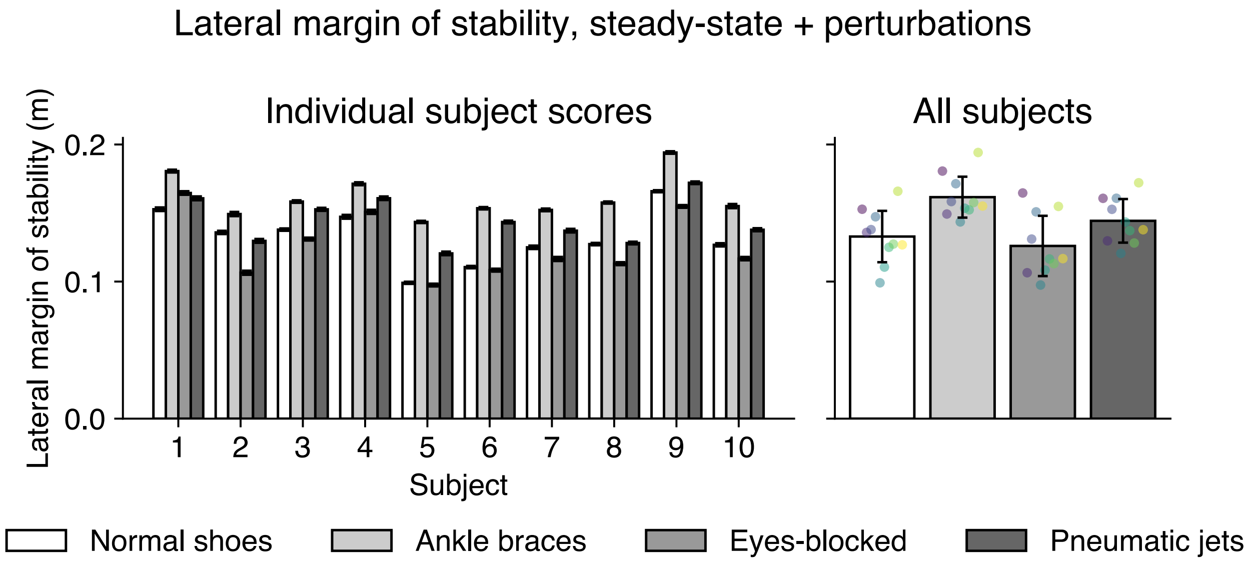

Lateral margin of stability, steady-state + perturbations
Individual subject scores
All subjects
Lateral margin of stability (m)
0.2
0.1
0.0
1
2
3
4
5
6
7
8
9
10
Subject
Normal shoes
Ankle braces
Eyes-blocked
Pneumatic jets

F

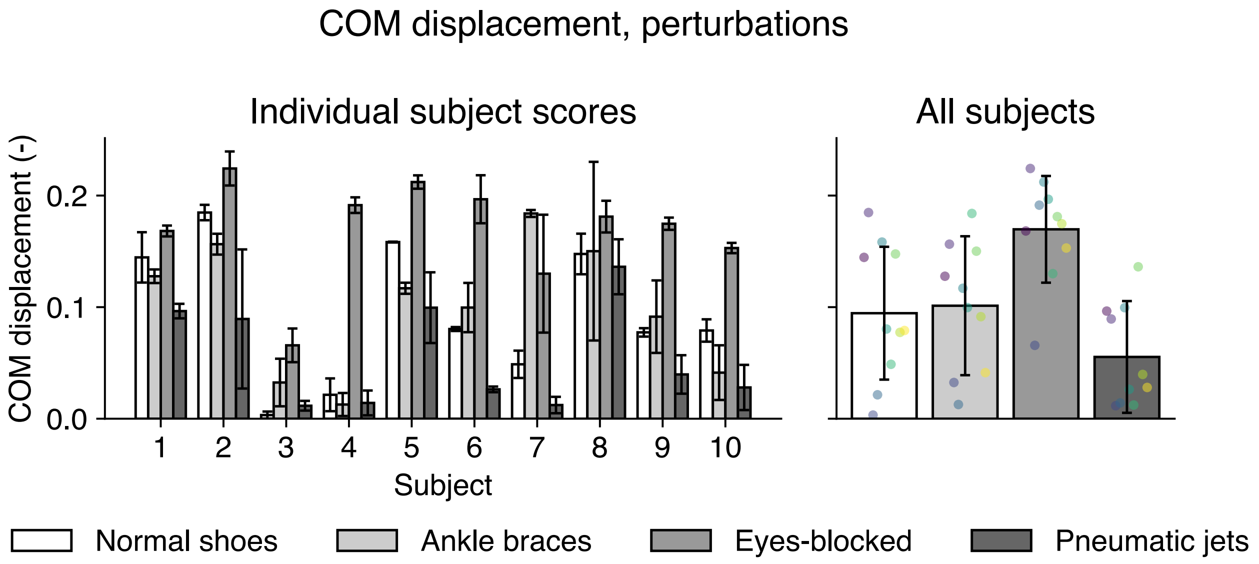

COM displacement, perturbations
Individual subject scores
All subjects
COM displacement (-)
0.2
0.1
0.0
1
2
3
4
5
6
7
8
9
10
Subject
Normal shoes
Ankle braces
Eyes-blocked
Pneumatic jets

Fig. S3. Plots showing scores for each subject across all four artificial impairment conditions using steady-state + perturbation recovery data for all metrics except COM displacement, which uses only perturbation recovery data. Plots in the left columns show individual scores and plots in the right column show scores averaged across subjects, grouped by impairment condition. Scores are averaged over 1000 bootstrap samples for all metrics except for Lyapunov exponent, which uses 100 bootstrap samples. Error bars represent standard deviation of the distribution across all bootstrap samples. The right panels show mean metric values (± standard deviation) across all 10 subjects. Each dot represents the mean value for an individual subject, with consistent colors indicating the same subject across panels. Fig. S3A shows step width variability, Fig. S3B shows step time variability, Fig S3C shows foot placement accuracy, Fig. S3D shows Lyapunov exponent, Fig. S3E shows lateral margin of stability, and Fig. S3F shows center of mass (COM) displacement. Center of mass displacement uses perturbation recovery data only, no steady-state data is included.

Journal of Experimental Biology • Supplementary information

Step length analysis

A

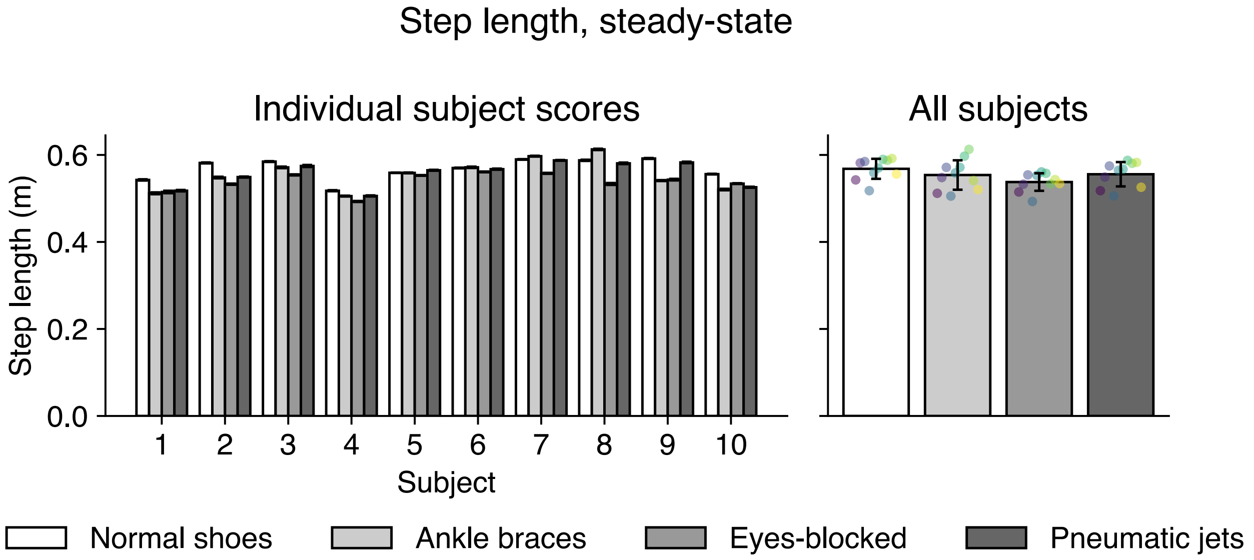

B

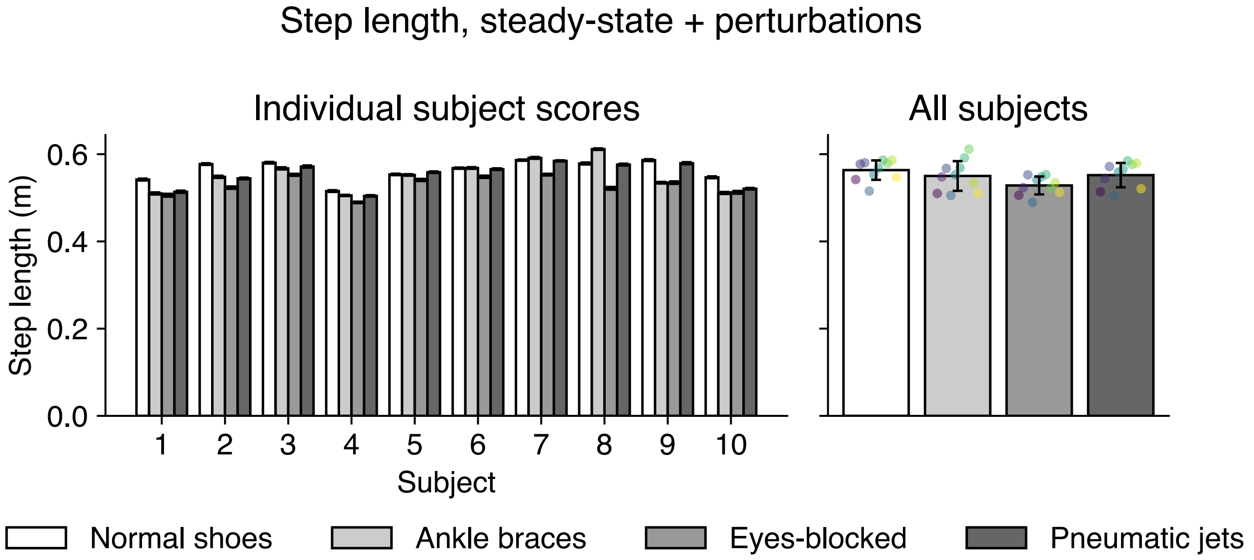

Fig. S4. Analysis of step length. The plots in the left column show individual subject scores and the plots in the right column show scores averaged across all subjects, grouped by impairment condition. Left bar plots represent the mean (± standard deviation) computed across 1000 bootstrap samples, right bar plots show mean metric values (± standard deviation) across 10 subjects. Each dot in right panels represent mean metric value for each subject, with consistent colors indicating the same subject across panels. Fig. S4A shows results from using steady-state data. Using subject-specific thresholds with steady-state data results in an impairment detection accuracy of 86%. Fig. S4B shows results from using steady-state plus perturbation recovery data. Using subject-specific thresholds with steady-state plus perturbation recovery data results in an impairment detection accuracy of 85%.

Journal of Experimental Biology • Supplementary information

Anterior margin of stability analysis

A

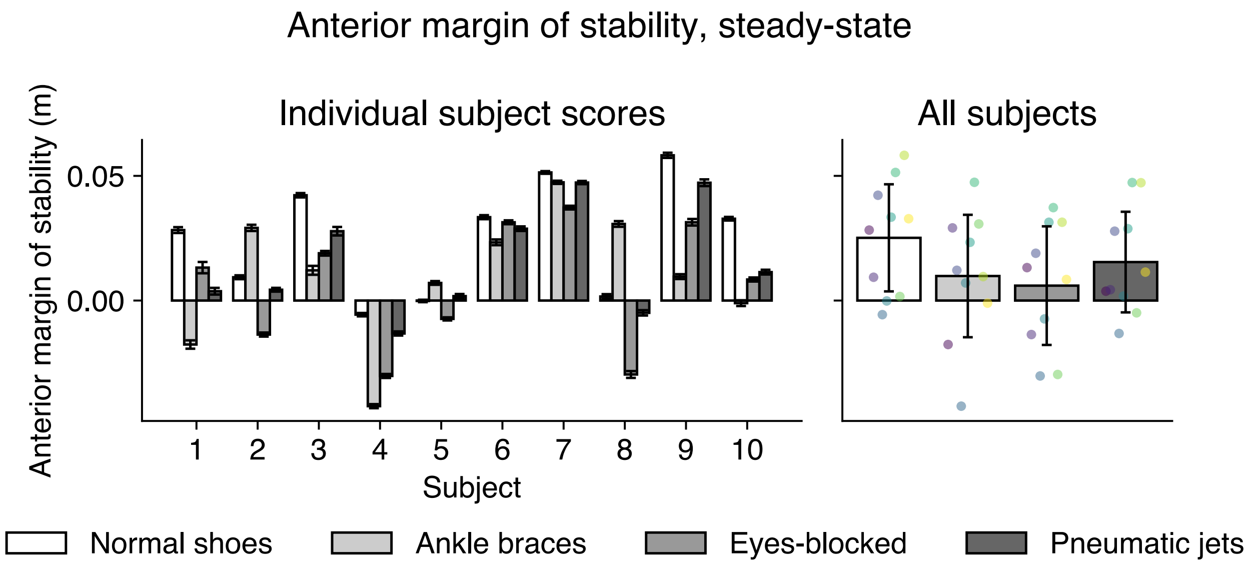

B

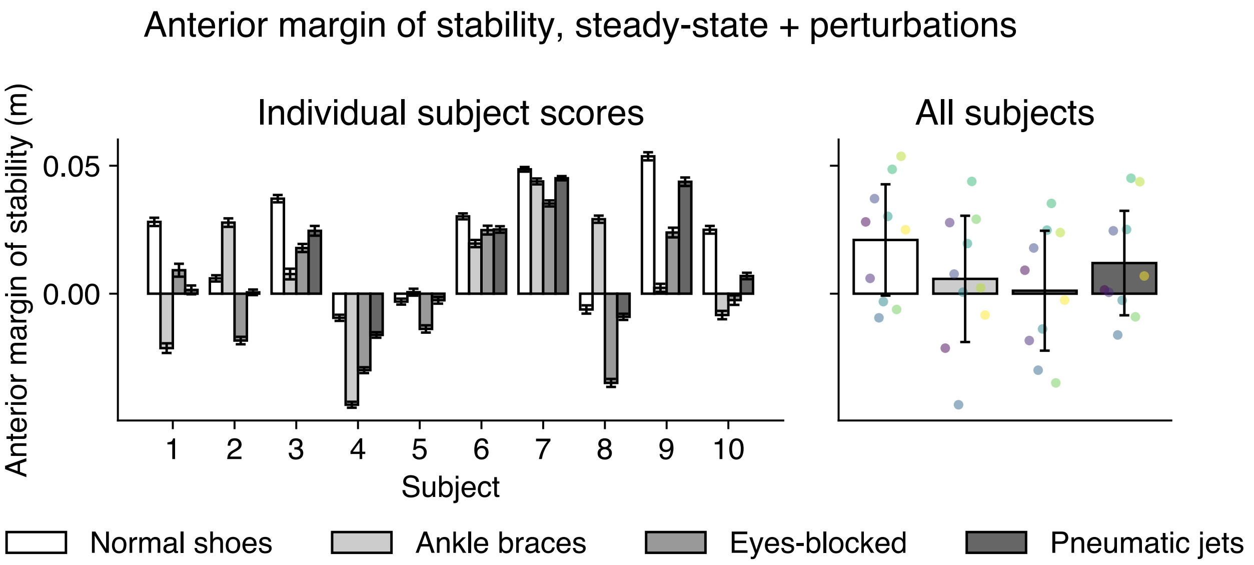

Journal of Experimental Biology • Supplementary information

Step width analysis

C

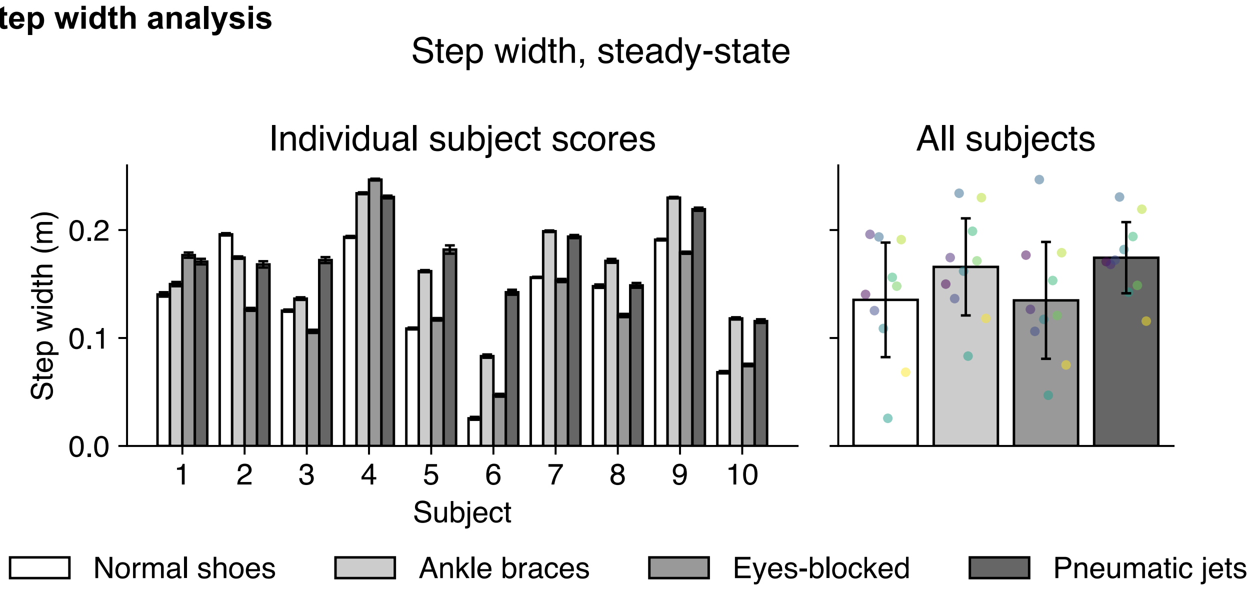

D

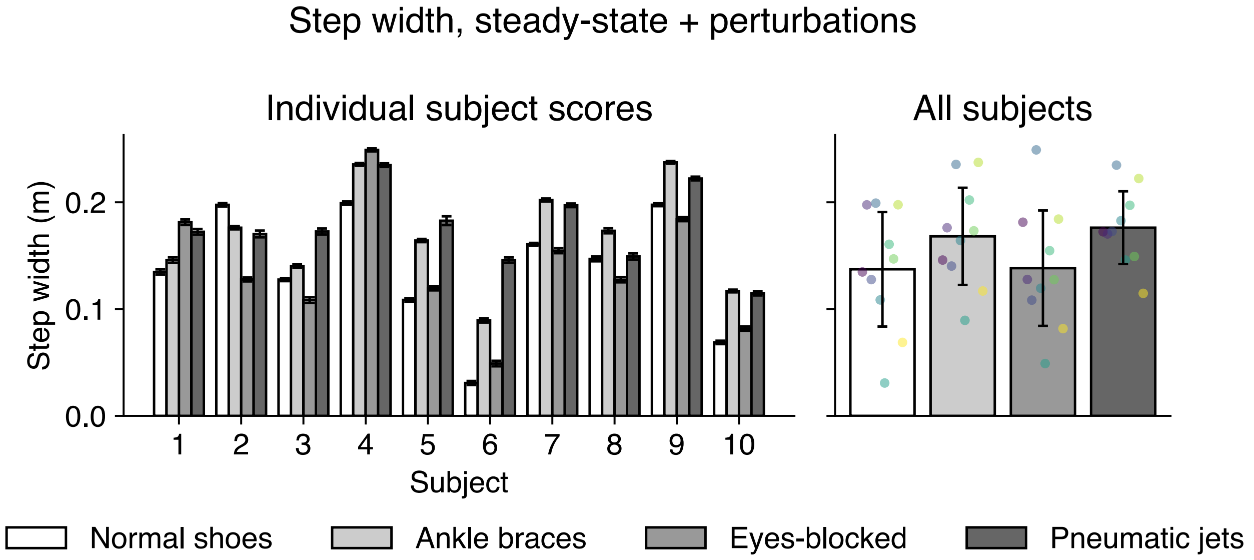

Fig. S5A-B. Analysis of anterior margin of stability. The plots in the left column show individual subject scores and the plots in the right column show scores averaged across all 10 subjects, grouped by impairment condition. Bar plots represent the mean across 1000 bootstrap samples and error bars represent standard deviation across bootstrap samples. Each dot in right panels represent mean metric value for each subject, with consistent colors indicating the same subject across panels. Fig. S5A shows results from using steady-state data. Using subject-specific thresholds with steady-state data results in an impairment detection accuracy of 13% when considering higher margin of stability to indicate impaired balance. Fig. S5B shows results from using steady-state plus perturbation recovery data. Using subject-specific thresholds with steady-state plus perturbation

recovery data results in an impairment detection accuracy of 12% when considering higher margin of stability to indicate impaired balance. Fig. 5C-D. Analysis of step width. The plots in the left column show individual subject scores and the plots in the right column show scores averaged across all subjects, grouped by impairment condition. Bar plots represent the mean across 1000 bootstrap samples and error bars represent standard deviation across bootstrap samples. Fig. S5C shows results from using steady-state data. Using subject-specific thresholds with steady-state data results in an impairment detection accuracy of 75%. Fig. S5D shows results from using steady-state plus perturbation recovery data. Using subject-specific thresholds with steady-state plus perturbation recovery data results in an impairment detection accuracy of 76%.

Journal of Experimental Biology • Supplementary information

Table S1. A. Summary of accuracy for each metric, movement data used, and detection threshold type without using bootstrapping. Fraction describes number of correct impaired balance detections over total number of detections. Steady-state plus perturbation recovery results calculated using both steady-state and perturbation recovery data for all metrics except center of mass displacement, which uses only perturbation recovery data (denoted by *). Materials and methods contain detailed description of calculations. Bootstrapped data was not used for this analysis. Table S1B-G. Logistic regression models for step width variability (**B**), step time variability (**C**), foot placement predictability (**D**), Lyapunov exponent (**E**), lateral margin of stability (**F**), and COM displacement (**G**) using steady-state data across 10 cross-validation folds. Table S6. Logistic regression models for using steady-state data across 10 cross-validation folds.

A

		Metric used					
Detection threshold	Data used	Step width var.	Step time var.	Foot place. accuracy	Lyapunov exponent	Lat. Margin of stability	COM disp.
Subject-specific	Steady-state	26/30	28/30	29/30	21/30	19/30	18/30
	Steady-state plus perturb.	21/30	20/30	23/30	19/30	21/30	15/30*
Cohort-based	Steady-state	20/40	25/40	28/40	20/40	24/40	19/40
	Steady-state plus perturb.	21/40	18/40	24/40	25/40	23/40	22/40*

B

Fold	Intercept	Coefficients	Threshold	Accuracy (%)	Sensitivity (%)	Specificity (%)
1	0.21	1.01	-0.21	50	33	100
2	0.22	1.04	-0.21	25	0	100
3	0.21	1.00	-0.21	75	100	0
4	0.20	0.99	-0.20	25	0	100
5	0.20	1.00	-0.20	75	67	100
6	0.17	0.86	-0.20	50	33	100
7	0.22	1.03	-0.21	25	0	100
8	0.18	0.92	-0.19	50	33	100
9	0.18	0.95	-0.19	50	33	100
10	0.28	1.24	-0.22	75	100	0

C

Fold	Intercept	Coefficients	Threshold	Accuracy (%)	Sensitivity (%)	Specificity (%)
1	0.39	1.47	-0.26	75	67	100
2	0.38	1.45	-0.26	75	67	100
3	0.43	1.56	-0.26	75	100	0
4	0.43	1.56	-0.27	25	0	100
5	0.39	1.50	-0.26	75	67	100
6	0.45	1.60	-0.28	50	33	100
7	0.42	1.53	-0.27	25	0	100
8	0.39	1.33	-0.30	75	67	100
9	0.39	1.46	-0.26	75	67	100
10	0.45	1.61	-0.28	75	100	0

D

Fold	Intercept	Coefficients	Threshold	Accuracy (%)	Sensitivity (%)	Specificity (%)
1	0.42	1.41	-0.30	100	100	100

2	0.46	1.49	-0.31	50	33	100
3	0.51	1.56	-0.32	75	100	0
4	0.43	1.43	-0.30	75	67	100
5	0.49	1.53	-0.32	75	100	0
6	0.47	1.50	-0.31	50	33	100
7	0.49	1.54	-0.32	50	33	100
8	0.42	1.41	-0.29	75	67	100
9	0.44	1.45	-0.30	75	67	100
10	0.52	1.59	-0.33	75	100	0

E

Fold	Intercept	Coefficients	Threshold	Accuracy (%)	Sensitivity (%)	Specificity (%)
1	0.03	0.36	-0.08	75	67	100
2	0.04	0.42	-0.09	50	67	0
3	0.06	0.50	-0.11	50	33	100
4	0.05	0.49	-0.11	25	0	100
5	0.03	0.38	-0.08	50	33	100
6	0.04	0.41	-0.09	50	67	0
7	0.05	0.46	-0.10	25	0	100
8	0.03	0.37	-0.09	75	100	0
9	0.06	0.56	-0.12	25	33	0
10	0.03	0.38	-0.09	75	100	0

F

Fold	Intercept	Coefficients	Threshold	Accuracy (%)	Sensitivity (%)	Specificity (%)
1	0.05	-0.44	0.11	100	100	100
2	0.06	-0.48	0.12	50	33	100
3	0.79	-0.57	0.14	50	67	0
4	0.08	-0.56	0.14	25	0	100
5	0.66	-0.52	0.13	25	0	100
6	0.08	-0.58	0.14	75	100	0
7	0.07	-0.52	0.13	75	100	0
8	0.49	-0.45	0.11	50	33	100
9	0.08	-0.56	0.14	50	67	0
10	0.04	-0.42	0.10	100	100	100

G

Fold	Intercept	Coefficients	Threshold	Accuracy (%)	Sensitivity (%)	Specificity (%)
1	0.010	0.21	-0.05	50	33	100
2	0.005	0.16	-0.03	50	33	100
3	0.007	0.18	-0.04	50	67	0
4	0.005	0.16	-0.03	25	0	100
5	0.009	0.20	-0.04	50	67	0
6	0.002	0.10	-0.02	50	33	100
7	0.010	0.21	-0.05	25	0	100
8	0.005	0.15	-0.03	75	67	100
9	0.001	0.05	-0.01	50	33	100
10	0.019	0.30	-0.06	50	67	0

Journal of Experimental Biology • Supplementary information
